# Direct Conversion of Ethanol to Ethyl Acetate by Dynamic Polyoxometalate/Carbon Nanohorn Electrocatalytic Interfaces

**DOI:** 10.1021/jacs.5c10817

**Published:** 2025-09-11

**Authors:** Simone Guazzi, Robin N. Dürr, Andrea Bogo, Elena Bassan, Goretti Arias-Ferreiro, Michele Cacioppo, Maurizio Prato, Marcella Bonchio

**Affiliations:** a Department of Chemical Sciences, 9308 University of Padova, Via Marzolo 1, Padova 35131, Italy; b INSTM, National Interuniversity Consortium of Materials Science and Technology, UdR, Padova I-35131, Italy; c Department of Chemical and Pharmaceutical Sciences, 9315 University of Trieste, Via Licio Giorgieri 1, Trieste 34127, Italy; d INSTM, National Interuniversity Consortium of Materials Science and Technology, UdR Trieste 34127, Italy; e Center for Cooperative Research in Biomaterials (CIC biomaGUNE), Basque Research and Technology Alliance (BRTA), Paseo de Miramón 194, Donostia/San Sebastián 20014, Spain; f Basque Foundation for Science, Ikerbasque, Bilbao 48013, Spain; g ITM-CNR, Istituto per la Tecnologia delle Membrane, UoS di Padova, Padova 35131, Italy

## Abstract

The electrosynthesis of ethyl acetate (EtOAc) by oxidative esterification of aqueous (up to 20% water) ethanol (EtOH) is performed by employing the tetraruthenate polyoxometalate [Ru_4_(μ-O)_4_(μ–OH)_2_(H_2_O)_4_(γ-SiW_10_O_36_)_2_]^10–^ (Ru_4_POM) electrocatalyst with carbon nanohorns (CNHs) as a heterogeneous support. This strategy involves a voltage-gated electro-adsorption of Ru_4_POM on CNH-modified glassy carbon anodes with favorable interfacial dynamics, maintained under electrocatalytic conditions. These conditions are reached through the continuous reconstruction of the organic/inorganic interface (catch-and-release), as probed by converging thermal, microscopic, and electrochemical analyses. In fact, control experiments reveal that both pristine and N-doped CNHs display a Ru_4_POM loading in the range 13–18 nmol mg^–1^ with a remarkable ∼100 mV onset potential anticipation and current enhancement in the range 400–700% compared to the homogeneous conditions. By adopting the “catch-and-release” protocol, electro-esterification of aqueous EtOH features long-term stability of the productive current in the mA range (*J*_chrono_ ≈ 2 mA cm^–2^ at +1.2 V vs Ag/AgCl probed up to 18 h), with Faradaic efficiencies, FE_EtOAc_, of >90%. This effect is attributed to the crucial role of CNHs hydrophobicity to control hydrolysis equilibria, thus outperforming the solution-phase behavior, which levels off at FE_EtOAc_ < 60%.

## Graphical abstract

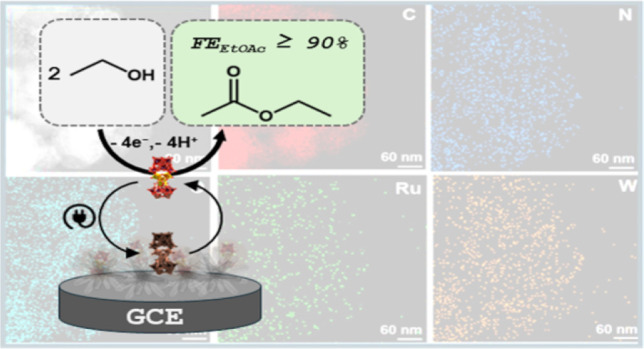

## 1 Introduction

The selective conversion of renewable biofuels into value-added products has a fundamental interest in the field of sustainable catalysis for green chemical synthesis.^1–3^ The annual global production of ethyl acetate (EtOAc), amounting to millions of tons, highlights a particularly relevant target. The conventional esterification of ethanol (EtOH) with acetic acid presents several challenges, including low selectivity and conversion of raw feedstocks, as well as the high energy cost associated with a multistep synthesis and purification process.^4,5^

Process intensification through tailored electrocatalytic methods can improve productivity and energy efficiency by strategically reducing the number of synthetic steps and integrating sequential reactions. This approach can also take advantage of alternative and renewable energy sources while enabling low-energy and selective reaction pathways. In this context, the design of selective electrocatalysts for the one-step electrocatalytic oxidation of aqueous EtOH (i.e., from bioethanol sources) to EtOAc is a key strategy. It allows for the combination of oxidation and esterification steps and paves the way for the use of renewable energy sources (e.g., solar, wind) to power the required cell voltage.^1,2,6^

The direct electrosynthesis of EtOAc from aqueous bioethanol presents several formidable challenges: (i) the competitive water oxidation reaction, which evolves oxygen and generates corrosive reactive oxygen species (ROS);^3,7–9^ (ii) the risk of unselective oxidation pathways leading to complete conversion of EtOH to CO_2_, as commonly observed in ethanol fuel cells;^10^ (iii) the need for precise mechanistic control over reactive intermediates and kinetics to facilitate the combined oxidation/esterification turnover;^11–13^ (iv) the minimization of reverse EtOAc hydrolysis through the use of tailored hydrophobic environments; (v) the engineering of electroactive molecular/cluster junctions to enhance electrode performance, ensure long-term stability, and optimize the activity–selectivity trade-off.^14–18^

To address these issues, the tetraruthenate polyoxometalate [Ru_4_(μ-O)_4_(μ–OH)_2_(H_2_O)_4_(γ-SiW_1__0_O_3__6_)_2_]^10^^–^ (Ru_4_POM), in addition to its well-documented activity as a water oxidation catalyst (WOC),^19–23^ has also been reported as an electrocatalyst for the partial ethanol oxidation reaction (EOR), as well as other Ru-oxo complexes, producing a mixture of aldehyde and acetic acid in dilute or acidified aqueous solutions.^17,24–26^ Notably, the electrocatalytic activity of Ru_4_POM is significantly enhanced when incorporated into composite materials assembled with highly conductive carbon nanostructures (CNS), which offer a high surface area and N-based, positively charged anchoring domains. Indeed, the polyanionic nature of this molecular cluster has been effectively exploited for immobilization on cationic carbon-based supports, including polymeric composites,^17,27^ carbon nanotubes,^20,28–30^ and graphene.^31–33^

Building on this platform,^27–29^ the present work focuses on leveraging Ru_4_POM to promote the direct oxidative esterification of EtOH to EtOAc under electrocatalytic conditions. In this system, the Ru_4_POM cluster plays a dual role as both an active solution-phase electrolyte and catalyst reservoir, and a heterogeneous single-cluster catalyst (SCC) immobilized on nanostructured carbon-based electrodes, boosting both catalytic current and selectivity for EtOAc production at the organic–inorganic heterojunction.^34^

Our findings highlight a continuous restructuring of the active interface, driven by electro-adsorption of Ru_4_POM onto tailored nanocarbon materials under anodic bias. This process significantly enhances the electrosynthetic performance. In this study electrocatalysis by Ru_4_POM was first optimized in homogeneous solution (protocol A in the Equipment and Methods section of the Supporting Information), then investigated under heterogeneous conditions using tailored carbon nanohorns (CNHs) supports (protocol B in the Equipment and Methods section of the Supporting Information), and ultimately integrated into a hybrid homogeneous/heterogeneous setup under electro-adsorption conditions (protocol C in the Equipment and Methods section of the Supporting Information). This configuration enables on-surface ″catch-and-release″ dynamics of POM clusters, offering operando healing of defects and mitigating catalyst deactivation due to leaching (see the Electro-adsorption section of the Supporting Information).

Among CNS suitable for electrocatalysis, dahlia-shaped CNHs are particularly attractive due to their dual advantages: the excellent electrical conductivity afforded by graphene-like connectivity across multicone junctions and their high surface area and nanoporosity.^35,36^ This unique structure fosters efficient Ru_4_POM adsorption and provides a hydrophobic environment expected to counterbalance EtOAc hydrolysis. To this end, novel Ru_4_POM–CNHs composites were prepared using both pristine CNHs (p-CNHs) and protonated nitrogen-doped CNHs (N-CNHs) as a positively charged network for POM anchoring through electrostatic attraction.

We demonstrate that Ru_4_POM immobilization on CNHs is effectively driven by electro-adsorption, facilitated by applying a positive potential to the nanostructured electrode within the electrochemical cell. Anodic polarization of the CNHs scaffold proves instrumental in situ, promoting polyanion migration across the nanocarbon interface at +500 mV applied bias (vs Ag/AgCl).

Significantly, the optimized Ru_4_POM electrocatalysis under voltage-gated CNHs dynamic confinement achieves: (i) record current densities for the direct oxidative esterification of aqueous EtOH (96%), with an average J_chrono_ = 1.66 mA cm^–2^ over 4 h chronoamperometry performed at +1.2 V vs Ag/AgCl, setting a 700% enhancement compared to Ru_4_POM homogeneous electrocatalysis, (ii) excellent long-term stability (up to 18 h), and (iii) unprecedented faradaic efficiencies for EtOAc (FE_EtOAc_ up to 90%), outperforming the solution-phase behavior, which levels off below 60% in aqueous EtOH.

## 2 Results and Discussion

### 2.1 Electrocatalytic Oxidation by Ru_4_POM in Aqueous EtOH

Cyclic voltammetry (CV) experiments of Ru_4_POM (40 μM) in aqueous EtOH (4% v/v H_2_O, υ_scan_ 100 mV s^–1)^ exhibit a major catalytic wave with onset at +1.13 V, current peak of 0.23 mA at +1.5 V vs Ag/AgCl, and negligible blank profile (Figure S4). Indeed, the current rise depends on the Ru_4_POM concentration in the range 40–200 μM, yielding a saturation plateau for [Ru_4_POM] > 100 μM, which identifies the ruthenate cluster as the competent electrocatalyst for EtOH oxidation (Figure S5).^17^

Analysis of the oxidation products has been performed after controlled potential electrolysis (CPE) at +1.2 V vs Ag/AgCl for up to 3.5 h (protocol A in the Equipment and Methods section of the Supporting Information), by combined high-performance liquid chromatography integrated with diode array detector (HPLC-DAD) and gas-phase chromatography (GC), according to a multiproduct calibration protocol against relevant standards (Figure S3, Tables S1 and S7). Under the conditions explored, EtOAc is formed with FE_EtOAc_ = 57 ± 3% while no acetaldehyde or the corresponding hemiacetal/acetal derivatives are detected, indicating a direct oxidative esterification pathway (calculation of FE for a 4-electron process is detailed in the SI). Indeed, no O_2_ nor CO were detected in the gas-phase (Figures S6–S8), confirming that the observed EOR selectivity builds on the noncompeting water oxidation and EtOH overoxidation.

The water content has a decisive role on the esterification outcome, as the FE_EtOAc_ values show a monotonic decrease upon water addition in the range 0–20% v/v, reaching up to 80 ± 4% in dry EtOH (Figure 1 and Figure S13). This observation is relevant for the processing of bioethanol solutions, which typically contain 4% v/v of H_2_O,^37^ while highlighting the importance of the catalyst microenvironment to enhance the esterification yield. This latter is emerging as a unique factor to target process intensification, as the conventional increase of the applied bias has a detrimental effect, with a steady reduction of FE_EtOAc_ ∼ 30% at +1.4 V (Figure 1 and Figure S16) and a progressive shift of the electrocatalysis toward competitive water oxidation at >+1.5 V vs Ag/AgCl (Figure S9).

**Figure 1:**
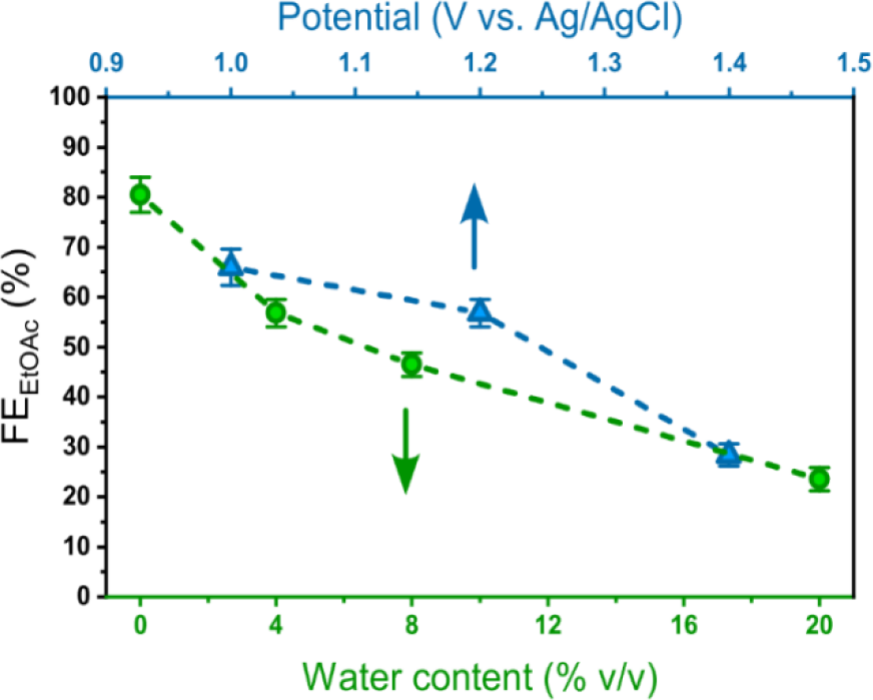
Dependence of FE_EtOAc_ on water content (green traces, applied potential: +1.2 V vs Ag/AgCl) or applied potential (blue traces, 4% v/v H_2_O).

In all cases, Ru_4_POM stability is assessed by combined UV–vis monitoring of the electrolyte solution and rinsing tests performed on new and spent working electrodes after CPE experiments. In particular, the UV–vis spectral features are consistent with a POM resting state of ∼88% in solution (Figure S8A), while consecutive CVs registered on a working electrode sample, recovered from the spent CPE experiment and washed with EtOH, show a steady current decrease at similar onset values, indicating a reversible absorption–desorption equilibrium occurring for Ru_4_POM at the electrode surface (Figure S8B).

Insights into the electrocatalytic mechanism are provided by control experiments, indicating that no EtOAc is detected under thermal, unwired conditions upon allowing Ru_4_POM (40 μM) to react with aqueous EtOH (4% H_2_O, 0.2 M LiClO_4_·3H_2_O) with or without addition of acetic acid (27 mM) (Figure S17A,B), ruling out acetic acid esterification as the relevant step. In line with literature studies,^17,38–42^ this evidence supports the reaction scheme reported in Figure 2, (see also Figure S18), including the following steps: (i) two-electron oxidation of EtOH to acetaldehyde; (ii) hemiacetal formation equilibria in EtOH; (iii) two-electron oxidation of the hemiacetal intermediate yielding EtOAc. The direct oxidative esterification to EtOAc is therefore promoted in EtOH media while hampered in dilute water solution, where the hemiacetal formation is disfavored and the cumulative 4-electron oxidation to acetaldehyde and acetic acid is the major pathway (Table S1). Furthermore, in our study, no formation of aldehydic intermediates is observed, which indicates that the kinetics of the hemiacetal transient is too rapid for the time scale of the experiment. Noteworthy, in analogous experimental conditions, electrocatalytic oxidation of aqueous methanol (MeOH) by Ru_4_POM yields methyl formate, with FE of 79 ± 5%, which is consistent with the favored hemiacetal formation (Figures S20–S22).

**Figure 2:**
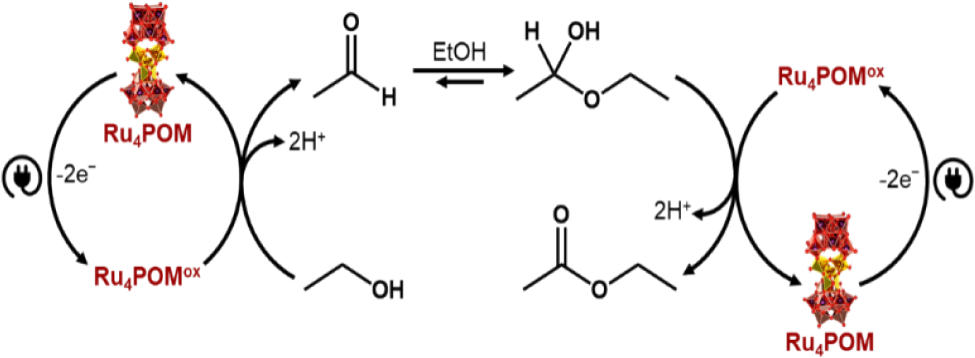
Schematic representation of the proposed mechanism for the electrocatalytic oxidation of EtOH and esterification to EtOAc performed in 2.5 mL of electrolytic solution (96% EtOH, 4% v/v H_2_O electrolytic solution, 0.2 M LiClO_4_, 40 μM Ru_4_POM) at +1.2 V vs Ag/AgCl applied bias (Equipment and Methods section of the Supporting Information).

### 2.2 Fabrication of Ru_4_POM@CNHs Electrocatalytic Interfaces

The anchoring of Ru_4_POM on nanocarbon supports provides a tunable electrode–electrolyte interface with built-in organic/inorganic heterojunctions for enhanced mass-transport, absorption, and diffusion within amphiphilic domains. In particular, a successful immobilization strategy of polyoxometalates on carbon-based supports can simply exploit complementary electrostatic interactions.^20,28,32,43^ Among all other CNSs, CNHs display excellent electron conduction, high surface area, electric field enhancement effect at the tips, and a versatile portfolio of surface derivatization protocols.^36,44–47^ In addition, N-doping of CNHs has been proposed as a way to improve conductivity and catalytic performance.^48–50^ Furthermore, the positively charged surface upon protonation of the pyridinic sites allows electrostatic interactions with the negatively charged POM catalyst. In this vision, the electrocatalyst microenvironment features a strong electrostatic adhesion by complementary charge interaction, together with a favorable interplay of electron–proton transfer domains, and a locally enhanced hydrophobic confinement for stabilization of intermediates and products generated during the electrocatalytic turnovers.

The synthesis of N-CNHs is accomplished with a solvothermal method, slightly modified from the literature protocol (Scheme S1).^51^ In a typical experiment, p-CNHs are ultrasonicated in water in the presence of hydrazine and ammonia for 1 h at room temperature and then heated to 150 °C in a Teflon-lined stainless steel autoclave for 4 h. The produced N-CNHs are collected by microfiltration, washed with Milli-Q water, rinsed with EtOH, and dried under vacuum. Analysis of the elemental composition by X-ray photoelectron spectroscopy (XPS, Figure S23, Tables S2–S4) shows that the resulting N-CNHs contain 95.30% C, 3.97% O, and 0.72% N atoms.

In particular, peak fitting of the N 1s high-resolution spectrum shows two components with maxima at 400.3 and 404.2 eV, attributable to backbone-inserted and oxidized nitrogen (such as pyridine *N*-oxide),^52^ respectively (Figure S23C).^53–55^ With respect to the p-CNH, Raman spectra of N-CNHs reveal a slight increase in the typical *I*_D_/*I*_G_ band ratio from 1.15 to 1.18, which is consistent with the N-heteroatom insertion,^56^ (Figure S30), while transmission electron microscopy (TEM) imaging shows the expected globular morphology of CNHs that is retained after the chemical treatment (Figure S28A).^57,58^

Anchoring of Ru_4_POM to either N-CNHs or p-CNHs (1.6 μmol of Na_10_Ru_4_POM per mg of N-CNHs) is promoted upon acid treatment (0.1 M HCl), as Z-potential analysis performed under acidic titration shows the mitigation of the negative surface charge and its reversion to positive values at pH < 4.0 (Figure S31, Scheme S1). Formation of positive domains upon protonation of N-CNHs is confirmed by XPS analysis (Figure S24).^54,59^ Acidification of p-CNHs shows a similar neutralization of the negative charge, likely due to protonation of carboxylate residues (Figure S31).^17,26^

Ru_4_POM@N-CNHs and Ru_4_POM@p-CNHs were collected by microfiltration, washed with 0.1 M HCl and EtOH, and finally dried. Loading of Ru_4_POM on the carbon nanostructure was confirmed by XPS, observing the appearance of the W 4f orbital (Figures S26A,C and S27A,E). These latter data were further corroborated by TEM with high-angle annular dark-field (HAADF) imaging and energy-dispersive X-ray spectroscopy (EDX) mapping, showing a homogeneous dispersion of the catalyst on the surface and edges of the CNHs scaffold (Figure 3 and Figure S28). Thermogravimetric analysis (TGA) indicates that both N-doped and p-CNHs display a similar Ru_4_POM loading in the range 13–18 nmol mg^–1^ (Figure S29A,B), thus pointing to a favorable interplay of surface interactions mediated by reactive defects (N- or O– terminals) with the redox-active POM.^28,30^

**Figure 3:**
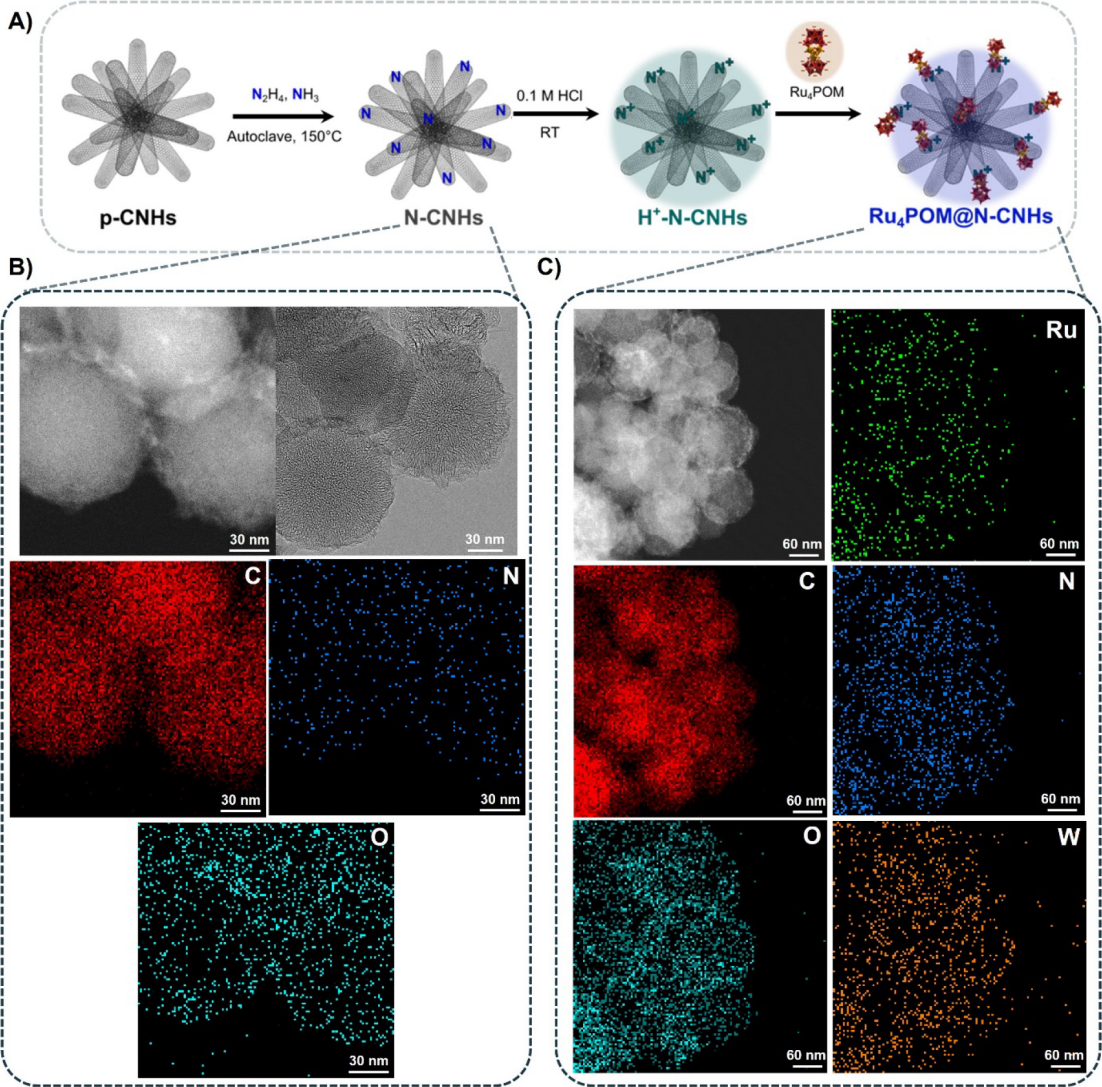
(A) Schematic representation of the Ru_4_POM@N-CNHs synthesis. (B, C) TEM-HAADF and TEM-EDX microscopy characterizations of N-CNHs and Ru_4_POM@N-CNHs.

### 2.3 Electrocatalytic Oxidation by Ru_4_POM@CNHs Hybrids in Aqueous EtOH

The leverage effect of the CNHs support for Ru_4_POM electrocatalysis is explored by drop-casting of Ru_4_POM@CNHs (MeOH, 1 mg mL^–1^) on glassy carbon electrodes (0.07 cm^2^ GCEs). The CV behavior shows a remarkable improvement of the Ru_4_POM@CNHs electrocatalytic performance with a ca. 100 mV onset potential anticipation (+1.04 V vs Ag/AgCl) and catalytic current rise overarching the Ru_4_POM solution-phase behavior (up to 3 vs 0.9 mA cm^–2^ at +1.2 V vs Ag/AgCl, Figure 4A). Blank controls show that all POM-free supports are catalytically inert (Figure 4A). The key role of the CNHs scaffold is highlighted by electrochemical impedance spectroscopy (EIS) showing very similar Nyquist plots and charge transfer resistance (*R*_CT_) of 700 Ω for both pristine and N-doped CNHs that integrate the active ruthenate electrocatalyst, at variance with the POM-free unreactive materials (Figure 4B).

**Figure 4:**
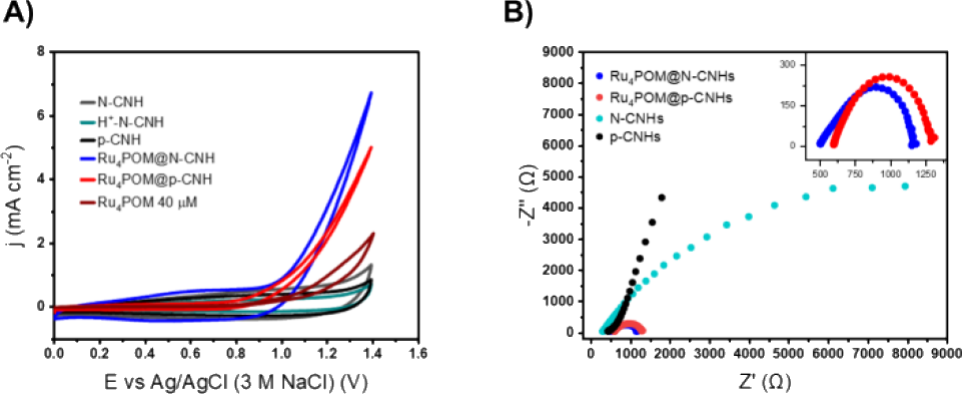
(A) CV of Ru_4_POM@N-CNHs (blue line), Ru_4_POM@p-CNHs (red line), N-CNHs (dark gray line), H^+^-N-CNHs (dark cyan line) and p-CNHs (black line), homogeneous Ru_4_POM (40 μM) (brown line); CV scan rate 100 mV s^–1^; (B) Nyquist plot of Ru_4_POM@N-CNHs (blue dots), Ru_4_POM@p-CNHs (red dots), N-CNHs (cyan dots), and p-CNHs (black dots) electrodes at +1.2 V vs Ag/AgCl; inset: zoom on Ru_4_POM@N-CNHs and Ru_4_POM@p-CNHs Nyquist plots. The experiments were performed in EtOH, H_2_O 4% v/v and LiClO_4_ 0.2 M electrolytic solution; RE: Ag/AgCl; CE: Pt wire.

Benchmark analysis, spanning diverse materials batches for N-CNHs and p-CNHs scaffolds, has been considered by evaluating the electrocatalytic behavior, the electrochemical surface area, and double-layer (EDL) capacitance (*C*_dl_) of the resulting Ru_4_POM@CNHs electrodes (Figures S32–S34). Our results indicate that a similar boosting effect is obtained with either N-CNHs or p-CNHs scaffolds, integrating Ru_4_POM with a similar loading as detected by TGA experiments (18–13 nmol mg^–1^) (Figure S29A,B).

The robustness of the Ru_4_POM@CNHs composite has been tested under CPE conditions (Figure S35), whereby major losses of the current density are observed in the range 78–61% over 4 h for both p-CNHs or N-CNHs scaffolds, likely due to a steady leaching of the Ru_4_POM catalyst (Table S10).^30,60^

Indeed, the working electrode has been rinsed and probed after use by consecutive CV experiments, showing a progressive desorption of the polyanionic cluster (Figures S8 and S36A–D)

Adsorption–desorption equilibria and their complex interplay are of fundamental importance for electrocatalytic reactions regulated by inner-sphere mechanisms and by the surface stabilization of reactants/intermediates.^61^ Therefore, the “catch-and-release” phenomena of Ru_4_POM@CNHs is expected to be tuned by (i) the applied anodic voltage with electric field hotspots at sharp tips/curvatures features of the nanocarbon scaffold; (ii) the Ru_4_POM concentration within the aqueous electrolyte; (iii) the time-domain of adsorption–desorption dynamics that ultimately regulate the electrocatalyst performance and selectivity.^62^

Building on these concepts, we envisage a voltage-gated electro-adsorption of molecular Ru_4_POM clusters on CNHs-modified GCE under applied positive bias (Figure 5).^63–65^ To this aim, drop-casting of N-CNHs or p-CNHs dispersions (1 mg mL^−1^ in methanol) on glassy carbon disks has been optimized for deposition of the CNHs scaffold on the GCE working electrodes, followed by immersion within a Ru_4_POM containing electrolyte solution (40 μM in 96% EtOH, 0.2 M LiClO_4_) while maintained under an applied potential of +0.5 V vs Ag/AgCl for 30 min. As a result of the liquid-electrode interfacial dynamics, electro-adsorbed Ru_4_POM@CNHs show a CV behavior that is superimposable to the conventional heterogeneous electrode and confirms the prominent electrocatalytic oxidation by confined Ru_4_POM on both N- or p-CNHs scaffolds (Figure 6A, Tables S7 and S8). Noteworthy, manipulation of the Ru_4_POM adsorption equilibria by the combined effect of the surface polarization and electrolyte saturation leads to a remarkable improvement of the *J*/*V* experimental profile that remains stable over 10 consecutive CV cycles (Figure S36C,D). Control experiments with no voltage-gated electro-adsorption step nor Ru_4_POM as an additive to the electrolyte display a continuous abatement of the current profile resulting from the favored Ru_4_POM release in solution (Figures S36A,B). The electro-adsorption step leads to a substantial increase of the double-layer capacitance of both p-CNHs and N-CNHs-based electrodes, with an EDL charge increase in the range 4 to 6-folds (*C*_dl_ increased from 5 to 30 μF for electro-adsorbed Ru_4_POM@p-CNHs and from 20 to 90 μF for electro-adsorbed Ru_4_POM@N-CNHs) (Figure S37). This behavior is consistent with the diffusion of the Ru_4_POM active clusters within the CNHs porous matrix that can be tuned by N-doping of the nanocarbon scaffold. Indeed, the electrochemically active surface area (ECSA) is estimated from the electrode *C*_dl_, yielding values up to 289 cm^2^ mg^−1^ for E-ads/Ru_4_POM@N-CNHs (see eqs 9 and 10 and Table S7 in the Supporting Information), which confirms the relevance of the electro-adsorption equilibria at the electrode/electrolyte interface (see the Electro-adsorption section in the Supporting Information).

**Figure 5:**
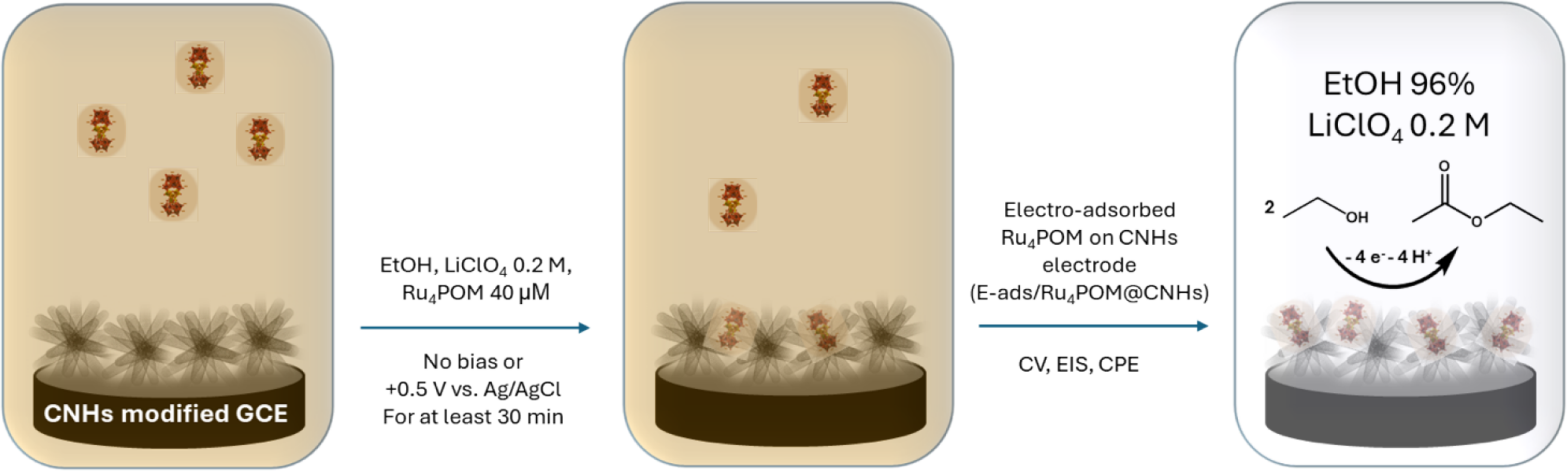
Scheme of the experimental setup for the synthesis and electrochemical characterization of electro-adsorbed Ru_4_POM on CNHs-modified GCE (E-ads/Ru_4_POM@CNHs).

**Figure 6:**
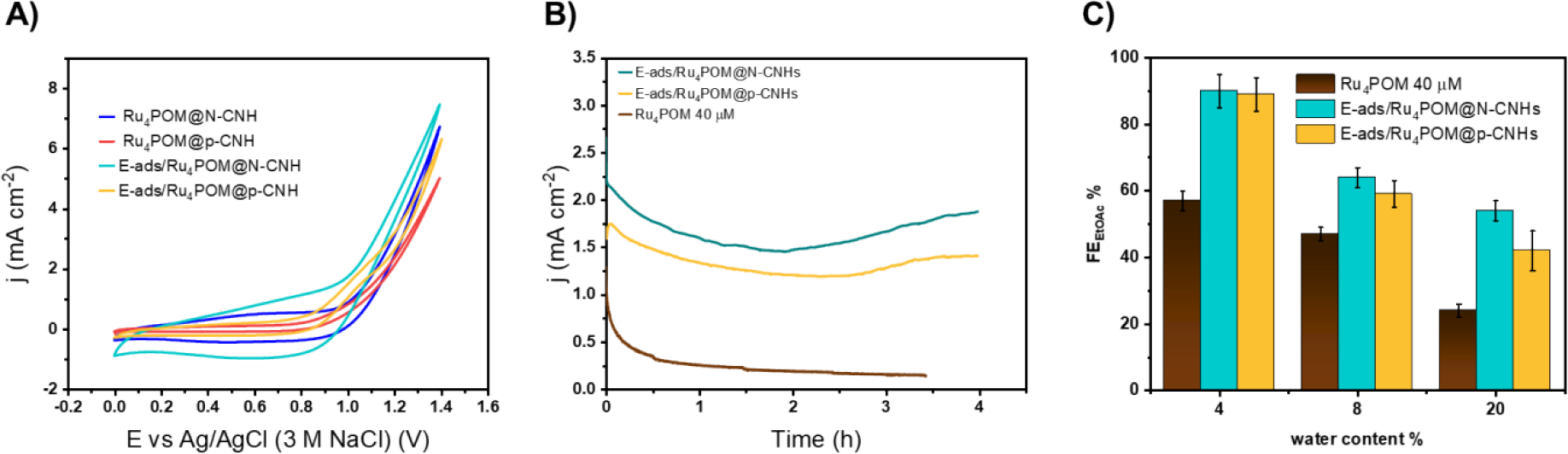
(A) CVs of conventional heterogeneous Ru_4_POM@N-CNHs (blue line), Ru_4_POM@p-CNHs (red line) electrodes performed in EtOH, H_2_O 4% v/v and LiClO_4_ 0.2 M electrolytic solution and superimposed CVs of electro-adsorbed analogs, E-ads/Ru_4_POM@N-CNHs (cyan line), E-ads/Ru_4_POM@p-CNHs (yellow line) performed in EtOH, H_2_O 4% v/v, LiClO_4_ 0.2 M with added Ru_4_POM 40 μM electrolytic solution, scan rate 100 mV s^–1^; (B) CPE at +1.2 V vs Ag/AgCl over 4 h of E-ads/Ru_4_POM@N-CNHs (cyan line) and E-ads/Ru_4_POM@p-CNHs (yellow line) in a solution of LiClO_4_ 0.2 M and Ru_4_POM 40 μM in EtOH 96%; as reference is reported the CPE of polished GCE in the same solution (brown line); (C) FE_EtOAc_ % vs water content v/v %: all the FE % were calculated after the CPEs. WE: bare glassy carbon disk (brown bars), E-ads/Ru_4_POM@N-CNHs-modified GCE (cyan bars) and E-ads/Ru_4_POM@N-CNHs-modified GCE (yellow bars); the experiments were performed in a solution of LiClO_4_ 0.2 M, Ru_4_POM 40 μM in EtOH 96, 92, and 80% respectively.

Electrocatalytic oxidation of aqueous EtOH was thus performed under “catch-and-release” conditions by E-ads/Ru_4_POM@CNHs electrodes during 4 h CPE experiments upon addition of Ru_4_POM (40 μM) within the electrolyte phase (Figure 6B and Figure S39). The continuous reconstruction of the CNHs-electrolyte interface guarantees a significant improvement of the current stability, approaching a 2 mA cm^–2^ plateau with long-term operation tested over 18 h (Figure S36F). In these experiments, the current response shows quasi-oscillatory dynamics, emerging from competing adsorption/desorption equilibria of Ru_4_POM that lead to a time-dependent distribution and coverage of active sites on the CNHs scaffold (E-ads/Ru_4_POM@CNHs trends in Figure 6B). On the contrary, a steady and irreversible deactivation is observed under conventional heterogeneous or homogeneous Ru_4_POM electrocatalysis (Figure 6B and Figure S36A–D).

It turns out that electrocatalysis by E-ads/Ru_4_POM@CNHs works with turnover frequency (TOF) values up to 1.47 s^–1^ and mass activity (MA) in the range 2572–2271 A g^–1^ (eqs 4–8 and Table S7 in the Supporting Information), based on the Ru_4_POM loading (nmol mg^−1^) as determined by TGA analysis (Figure S29). In particular, MA values are normalized on the mass of the catalytically active Ru_4_POM core ([Ru_4_(μ-O)_4_(μ–OH)_2_(H_2_O)_4_]^6+^ MW = 574.38 g mol^−1^), thus providing a direct analysis of the ruthenate sites' intrinsic activity compared to classical RuO_2_-based electrocatalysts (MA_RuO2_ = 34 A g^−1^ see benchmarking references in the Supporting Information).

By adopting the “catch-and-release” protocol, electro-esterification of aqueous EtOH proceeds with FE_EtOAc_ up to 92% with similar selectivity using either the N-CNHs or p-CNHs supports. In particular, the selectivity profile depends on the electrolyte water content in the range 4–20% (Figure 6C, Table S10). In all cases, the hydrophobic CNHs environment is instrumental in shielding the EtOAc product against hydrolysis, thus maximizing the process selectivity. Indeed, homogeneous electro-esterification by Ru_4_POM proceeds with FE > 80% in absolute EtOH albeit with low current densities (Table S10).

## 3 Conclusions

Confinement of the Ru_4_POM electrocatalyst within CNHs as both pristine or N-doped derivatives, provides highly selective, efficient, and robust electrocatalytic interfaces with unprecedented performances in the electrosynthesis of EtOAc from aqueous EtOH (Tables S7–S10). The unique properties of CNHs, including their conductivity, high surface area, nanoporosity, and hydrophobic character, are instrumental in leveraging high mass activity for the ruthenate cluster (MA > 2500 A g^−1^) applied to the direct oxidative esterification with FE_EtOAc_ > 90% using 96% EtOH. This protocol is amenable to the processing of bioethanol aqueous phases under an electrocatalytic setup by a favorable interplay of lowering the electrode resistance while shaping the active site environment within hydrophobic nanochannels that can prevent the reverse ester hydrolysis. Manipulation of the Ru_4_POM adsorption/desorption equilibria under voltage-gated conditions leads to the operando reconstruction of the Ru_4_POM@CNHs interface with long-lasting performance in the mA range probed over 18 h chronoamperometry (Figure S38). These results open the way to innovative electrosynthetic protocols that build on the synergistic effects arising from interfacial processes at the solid–liquid boundary.

## Supplementary Material



## Notes

Synthetic procedures of the materials; equations for calculating FE, mass activity, TOF_average_, TON, and catalyst loading; HPLC-derived calibration curves for EOR and methanol oxidation products; electrochemical characterization of the homogeneous and heterogeneous catalyst (CV, CPE, ECSA, and EIS), including control experiments and HPLC chromatograms of the electrolyte; additional characterization of supports and catalysts (UV–vis, zeta potential, TGA, Raman, XPS, and TEM) (PDF)

## ‡

Department of Chemistry and Applied Biosciences, ETH Zürich, Vladimir-Prelog-Weg 1–5/10, 8093 Zürich, Switzerland

## ∥

Department of Biological, Chemical and Pharmaceutical Sciences and Technologies (STEBICEF), University of Palermo, Viale delle Scienze, Ed. 17 – Stanislao Cannizzaro, 90128 Palermo, Italy
