# Supplementary material for: Direct Conversion of Ethanol to Ethyl Acetate by Dynamic Polyoxometalate/Carbon Nanohorn Electrocatalytic Interfaces
Source: J Am Chem Soc. 2025 Sep 11;147(38):34840–8. doi: 10.1021/jacs.5c10817 (PMC12464969; doi:10.1021/jacs.5c10817)
Supplement: Supplementary file 1 [file ja5c10817_si_001.pdf]

Supporting information for:

Direct Conversion of Ethanol to Ethyl Acetate by Dynamic Polyoxometalate/Carbon Nanohorns Electrocatalytic Interfaces

Simone Guazzi,[a,†] Robin N. Dürr,[a,‡,†] Andrea Bogo,[b,†] Elena Bassan,[a,†] Goretti Arias-Ferreiro,[c] Michele Cacioppo,[b,c,‖] Maurizio Prato[b,c,d,]* and Marcella Bonchio,[a,e]*

[a] Department of Chemical Sciences, University of Padova, Via Marzolo 1, 35131 Padova, Italy, INSTM, National Interuniversity Consortium of Materials Science and Technology, UdR Padova

[b] Department of Chemical and Pharmaceutical Sciences, University of Trieste, Via Licio Giorgieri 1, 34127 Trieste, Italy, INSTM, National Interuniversity Consortium of Materials Science and Technology, UdR Trieste

[c] Center for Cooperative Research in Biomaterials (CIC biomaGUNE), Basque Research and Technology Alliance (BRTA), Paseo de Miramón 194, 20014 Donostia/San Sebastián, Spain

[d] Basque Foundation for Science, Ikerbasque, 48013 Bilbao, Spain

[e] ITM-CNR, Istituto per la Tecnologia delle Membrane, UoS di Padova, 35131 Padova, Italy

* Email: mprato@cicbiomagune.es

* Email: marcella.bonchio@unipd.it

† S.G., R.N.D., A.B, E.B. contributed equally to this work.

Contents

Experimental section 2
Materials 2
Equipment and methods 2
Determination of faradaic efficiencies (FE) 4
Calculation of mass activity (MA), turnover numbers and frequency (TON, TOF) and of Electrochemical Active Surface Area (ECSA) for the optimized electro-adsorbed Ru_4POM@CNHs systems (protocol C) 5
Calculation of the turnover number (TON) 5
Calculation of turnover frequency (TOF) 6
Calculation of ECSA 6
Synthetic procedures 6
Ru_4POM catalyst 6
N-doped carbon nanohorns (N-CNHs) 6
Anchoring of Ru_4POM on p- and N-CNHs 6
Supporting figures 7
HPLC chromatographs of reference compounds 7
Homogeneous ethanol oxidation by Ru_4POM 8
Homogeneous methanol oxidation by Ru_4POM 15
XPS characterization of CNHs and Ru_4POM@CNHs hybrid materials 17
Transmission electron microscopy (TEM), thermogravimetric analysis (TGA), Raman spectroscopy and Zeta potential of CNHs and Ru_4POM@CNHs hybrid materials 24
Electrochemical surface area estimation by double layer capacitance 26
Electro-adsorption protocol for *in-situ* activation of the Ru_4POM@CNHs interface. 29
References 35

Experimental section

Materials

Lithium perchlorate trihydrate, absolute anhydrous ethanol (purity ≥ 99.9%) hydrochloric acid (puriss. p.a., ACS reagent, ≥ 37%), hydrophilic PTFE filter (Omnipore, 0.1 µm), denatured ethanol (95%), methanol (puriss. p.a., ACS reagent, ≥ 99.8%), bioethanol (96%, EMPROVE® EXPERT, Ph. Eur., BP, ChP), ammonium hydroxide (NH_4OH, ACS reagent, 28.0-30.0%), sulfuric acid (H_2SO_4, ACS reagent, 95.0-97.0 %), hydrogen peroxide (H_2O_2, 35%), and potassium hydroxide (KOH, ACS reagent ≥ 85%) were purchased from Merck and used without further purification. Potassium chloride (KCl, ≥ 98%, Sigma-Aldrich) was dried before used in an oven at 90°C for a couple of hours. Hydrazine monohydrate ($N_2H_4 \cdot H_2O$, ≥ 98%) and hydrochloric acid (HCl, 0.1 N TitriPUR® Reag. Ph. Eur., Reag. USP) were provided by VWR and also used without further purification.
Milli-Q® water for the electrochemical experiments was obtained using a Millipore apparatus, equipped with 0.22 µm filters. The glassy carbon disk (ItalSens) was supplied by PalmSens BV (GA Houten, The Netherlands). The Pt wire and Ag/AgCl electrode (BASi) was provided by Merck. Single Wall Carbon Nanohorns (CNHs, Dahlia-like, 3-4 nm diameter, 30-50 nm length) were purchased from Carbonium s.r.l. (Padova, Italy) and produced according to a patented method by direct graphite evaporation in Ar flow, without any catalyst.[1,2] Ultrapure fresh Milli-Q® water obtained by a Millipore water purification system (>18 MΩ Milli-Q, Millipore) was used in the synthesis and characterization regarding the CNHs.

Equipment and methods

Electrochemical setup: Electrochemical experiments cyclic voltammetry (CV) controlled potential electrolysis (CPE) and electrochemical impedance spectroscopy (EIS) were carried out with Metrohm Autolab potentiostat PGSTAT302N equipped with a FRA32M module (controlled with the software Nova 2.1.6) in a three electrode cell (Vol: 3-4 mL) at room temperature using air-equilibrated ethanol (96 %), Milli-Q water (4 %) and $LiClO_4 \cdot 3H_2O$ (0.2 M), unless otherwise stated. A Pt wire was used as the counter electrode (CE), as the working electrode (WE) a glassy carbon (GC) rod (diameter: 7 mm) was used for CPE while a GC disk (diameter: 3 mm) was used for CV and an Ag/AgCl (3 M NaCl) electrode as the reference electrode (RE), which was measured to be −388 mV vs. Fc/Fc^+ in the electrolyte. For homogeneous catalysis the working electrode was polished with a diamond paste (1 µm), rinsed with water, ultrasonicated for 15 min in water:acetone 1:1, rinsed with acetone and finally dried with N_2. For heterogeneous catalysis, the glassy carbon disk was polished with a 0.25 µm diamond paste, rinsed with water and methanol, ultrasonicated for 1-2 min in a water:methanol mixture, rinsed with water and finally dried with N_2.

To electrochemically investigate the supports with and without immobilized $[Ru_4(\mu\text{-}O)_4(\mu\text{-}OH)_2(H_2O)_4(\gamma\text{-}SiW_{10}O_{36})_2]^{10-}$ (Ru_4POM), an ink was formulated by mixing the compound (p-CNHs, N-CNHs, H^+-N-CNHs, Ru_4POM@p-CNHs, Ru_4POM@N-CNHs) in methanol with a mass concentration of 1 mg mL^{-1}, followed by ultrasonication for 1 h. 2.5 µL of the so-formed ink were drop-casted twice on the previously polished GC disk. In total 10 µL were drop-casted on a GC disk. Between each drop and before the electrochemical analysis, the solvent was completely evaporated. Onset potentials were determined as the intercept between the baseline current and the slope of the catalytic wave.

The potentials of the entries reported in **Table S7** and **S8** were converted from Ag/AgCl and reversible hydrogen electrode (RHE) to standard hydrogen electrode (SHE) using the following equations:

$$E_{\mathrm{SHE}} = E_{\mathrm{Ag\backslash AgCl}} + 209\ \mathrm{mV} \qquad (1)$$

$$E_{\mathrm{SHE}} = E_{\mathrm{RHE}} - (0.059 \times \mathrm{pH}) \qquad (2)$$

Cyclic Voltammetry (CV): To characterize the activity towards the ethanol oxidation reaction (EOR) cyclic voltammetry was employed between 0 – +1.4 V vs. Ag/AgCl or 0 – +2.0 V vs. Ag/AgCl with a scan rate of 100 mV s^{-1} and no *iR* compensation. Current density (j, mA cm^{-2}) was calculated normalizing the measured current (i) for the electrode geometric area (0.07 cm^{-2})

Controlled Potential Electrolysis (CPE): For detecting and quantifying the EOR reaction products of, CPE was carried out at +1.2 V vs. Ag/AgCl using an undivided three electrode cell set-up. Specific conditions refer to the three adopted protocols (A-C) and are described as follows:

(A) under homogeneous (solution-phase) conditions, electrocatalytic oxidation of ethanol was carried out with a polished glassy carbon electrode rod (GCE 2 cm^2) as working electrode, a Pt wire as counter electrode (H_2 evolution counter electrode process, see Figure S7) and an Ag/AgCl (3M NaCl) as reference electrode. All experiments were performed in 2.5 mL of electrolytic solution (EtOH, 0.2 M $LiClO_4$, 40 µM Ru_4POM and variable v/v percentages, %, of water as specified for every experiment) for up to 3.5 h under magnetic stirring conditions. The homogeneous electrocatalytic oxidation of methanol was carried out in the same conditions but applying +1.4 V vs. Ag/AgCl for up to 4.5 h under magnetic stirring conditions.

(B) Under heterogeneous conditions, a $Ru_4POM@CNHs$ modified glassy carbon electrode disk ($0.07\ cm^2$) was used as working electrode in 2.5 mL of the electrolytic solution (96% EtOH, 4% H2O v/v, 0.2 M $LiClO_4$) without stirring.

C) Under electro-adsorbtion conditions, a CNHs modified glassy carbon electrode disk (GCE $0.07\ cm^2$) was used as working electrode in 2.5 mL of electrolytic solution (EtOH, 0.2 M $LiClO_4$, 40 μM Ru_4POM and variable v/v percentages, %, of water as specified for every experiment) for up to 18 h without stirring (see also the specific session on the Electro-adsorption protocol for *in-situ* activation of the $Ru_4POM@CNHs$ interface).

Electrochemical Impedance Spectroscopy (EIS). To investigate changes in the charge transfer resistance (R_{CT}) among the materials, EIS was employed at +1.2 V vs. Ag/AgCl with frequencies from 200 kHz to 100 mHz, 10 frequencies per decade and an alternating voltage amplitude of 10 mV. Lissajous plots were recorded to verify the linearity of the I-V curve in the applied voltage window and Kramers-Kronig test of the software was used for further validation of the data. The EIS results were modeled with an Electrical Equivalent Circuit composed of a Voigt circuit (a resistor and a capacitor connected parallel, representing the charge-transfer resistance (R_{ct}) and the electrochemical double layer capacitance (C_{dl}), respectively) plus a solution resistance (R_s) serially connected with the Voigt circuit. R_{ct} was calculated from the Nyquist plot subtracting the value at the intersection of the semi-circle with the Z' axis at high frequencies (R_s) to the value at the intersection of the semi-circle with the Z' axis at low frequencies ($R_{ct} + R_s$).

Electrochemical Active Surface Area (ECSA): ECSA values for electro-adsorbed $Ru_4POM@CNHs$ materials were estimated from the double layer capacitance (Cdl) via cyclic voltammetry (CV) measurements, according to the equation ECSA= Cdl/Cs where Cs refers to the specific capacitance of CNH standards on a unit surface area. Here, Cs values of 11.4 and 31.1 $\mu F\ cm^{-2}$ have been considered respectively for pristine (p-CNHs) and N-doped (N-CNHs) materials as references for ECSA calculations (see equation 9). [3]

C_{dl} determination was performed by CV experiments in a non-faradaic region (0 – +0.2 V vs. Ag/AgCl) where the electrodes were hold at +0.1 V vs. Ag/AgCl for 2 min for equilibration and subsequently cycled at various scan rates (20, 40, 60, 80, 100 mV/s, Fig. S37). Then, the double layer capacitance (Cdl) was assessed from the slope of the linear regression between the current density differences ($i_{cap} = \Delta j/2 = (ja-jc)/2$) in the middle of the potential window of CV curves versus the scan rates (+0.1 V vs Ag/AgCl, Fig. S37).

UV-Visible Spectroscopy (UV-Vis): UV-Vis absorption spectra were recorded using a UV-Vis Cary 50 spectrophotometer and Hellma Analytics quartz cuvettes with an optical path of 10 mm.

High-Performance Liquid Chromatography (HPLC): HPLC chromatograms were obtained by means of a reverse-phase column (stationary phase: Phenomenex C-18; column length: 250 mm; internal diameter: 4.6 mm; particle size: 5 µm) with an isocratic mobile phase containing acetonitrile (65%) and water (35%) (flow rate: $0.6\ mL\ min^{-1}$) at 40°C. Calibrations by use of commercial standards were performed as described in the next section. In the chromatograms the absorbance at 210 nm over the retention time is displayed.

Micro gas chromatography (micro-GC): Micro-GC chromatographs were obtained using an Agilent 990 Micro-GC instrumentation employing a Molsieve 5Å (Agilent) column with argon as the carrier gas and a thermal conductivity detector (injection volume: 10 µL; sampling time: 10 s). Peaks' attribution was performed by comparison with commercial standards. The collected headspace volume is reintroduced in the in-line sealed cell after the analysis in order to minimize pressure loss inside the reactor.

Thermogravimetric Analysis (TGA): For TGA a TGA Q5000 V3.17 from TA Instruments controlled by the Advantage for QSeries software was used. The measurement was conducted in air with a flow rate of $25\ mL\ min^{-1}$. The samples were equilibrated in a Pt pan at 100 °C until a stable signal was obtained and then heated with $10\ °C\ min^{-1}$ to 950 °C ($Na_{10}Ru_4POM$, p-CNHs, N-CNHs and $Ru_4POM@p$-CNHs) or to 700 °C ($Ru_4POM@N$-CNHs). The data was exported with TA Instruments Universal Analysis 2000 Version 4.5A.

pH Measurement: The pH was measured with a Metrohm 827 pH lab meter, calibrated at pH 4 and pH 7.

Zeta-potential measurement: Zeta-potential was measured by electrophoretic light scattering, following a slightly modified, published procedure.[4] The titrating solutions of HCl, KOH and the water used to prepare the N-CNHs stock solution were filtered by using a PTFE syringe filter with a pore size of 0.45 µm (LLG-Syringe filters SPHEROS, LLG®Labware). All the solutions and suspensions for the Zeta-potential were prepared from Milli-Q® water (Millipore) with electrical resistance ≥18 MΩ. The CNHs (N-CNHs or p-CNHs, 0.3 mg) were dispersed in Milli-Q® (100 mL) and sonicated for 1 h by a US bath (Branson 2510 ultrasonic cleaner) to obtain a stock stable suspension (mass concentration: 3 mg/L). All the experiments were performed at 20 °C. To obtain the Zeta-potential at different pH values, the CNHs stock suspension was titrated with the HCl and KOH solutions, giving the titration curve. The titration was performed in the presence of KCl as electrolyte (10 mM), in order to adjust the conductivity of the stock solution. After preparing the sample, the suspension was injected into a capillary cell (DTS1070, Malvern). The cell was set on a Zetasizer NANO-ZS (Malvern instruments) equipped with a 4 mW 632.8 nm "red" laser and the Zeta-potential was measured by electrophoretic light scattering.

Raman spectroscopy: Raman spectra were acquired with a Renishaw instrument (model: inVia™ Reflex) equipped with green 532 nm/500 mW Cobolt CW DPSS (CDRH) laser, a 50× objective and WiRE 3.4 software. Raman spectra were calibrated with a silicon wafer reference (Silicon first order Raman scattering: 520.50 cm^{-1}). Samples were prepared by casting the powder on a microscope slide (PolysineTM, VWR) which was in turn allocated in the analyzer. The spectra were recorded at room temperature with 10 accumulations for each point keeping the laser power intensity at 0.5 %, to avoid sample damage. At least 10 spectra per sample were recorded on different areas of the sample in order to check the uniformity of the materials. After acquisition in 10 different points, the averaged spectra were normalized with respect to the G band. The intensity ratio of the D and G peaks was then calculated.

X-ray Photoelectron Spectroscopy (XPS): XPS experiments were performed in a Versaprobe III Physical Electronics (ULVAC) spectrometer with a monochromatic X-ray source (Aluminium Kα line of 1487 eV), calibrated using the 3d 5/2 line of Ag at 368.26 eV. Samples were mounted on isolating tape, and ion and electron neutralizers were used for charge compensation. Z-alignment was performed for optimal sample height prior to each sample measurement. Elemental quantification was done on survey scan with step energy of 0.5 eV, pass energy of 224 eV, and time per step 50 ms, while high resolution regions were acquired with step energy of 0.05 eV, pass energy of 55 eV, and time per step 50 ms. Data were analyzed by CasaXPS software (2.3.16 PR 1.6).[5] All spectra were charge corrected versus sp^2 carbon (C=C) at 284.5 eV. N 1s high-resolution spectra of N-CNHs, H^+-N-CNHs and Ru_4POM@N-CNHs were smoothed using Savitzky-Golay linear smoothing (width 5).

Transmission Electron Microscopy (TEM): TEM was conducted with a JEOL JEM-2100F UHR with a field emission gun operating at 200 kV. The instrument was equipped with a 4-megapixel CMOS camera (TVIPS TemCam-F216) and two scanning TEM detectors for bright field (BF) and high-angle annular dark-field (HAADF) imaging. For energy dispersive X-ray spectroscopy (EDX) mapping, an Oxford UltimMax detector was used. The samples were prepared by dispersing in absolute ethanol and ultrasonicating for 2 min. Then, the dispersion was drop-casted onto a lacey ultrathin carbon 400 mesh copper grid (Ted Pella) and left at air for drying. Image analysis and post processing was conducted with ImageJ Version 1.54f.[6] For better visibility the brightness and contrast were adjusted, and the HAADF images were additionally improved with the Enhance Local Contrast (CLAHE) function.

Determination of faradaic efficiencies (FE)

Calibration of ethyl acetate (EtOAc) was obtained by preparation of EtOAc solutions of known concentrations in EtOH (96%), H_2O (4%) and $LiClO_4 \cdot 3H_2O$ (0.2 M). The so-prepared samples were analyzed by HPLC and their peak's area at 210 nm was integrated. Each sample was prepared three times and statistical analysis of the results afforded the calibration curve shown in **Figure S1A**.

Calibration of acetic acid (AcOH) was obtained by preparation of AcOH solutions of known concentrations in EtOH (96%), H_2O (4%) and $LiClO_4 \cdot 3H_2O$ (0.2 M). The so-prepared samples were analyzed by HPLC and their peak's area at 210 nm was integrated. Each sample was prepared three times and statistical analysis of the results afforded the calibration curve shown in **Figure S1B**.

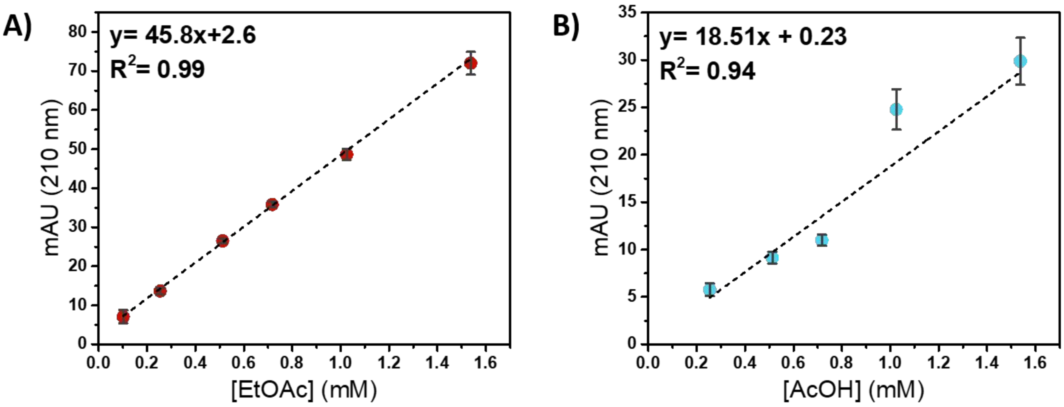

Figure S1. HPLC calibration curves for EtOAc (**A**) and AcOH (**B**) in EtOH ($LiClO_4$ 0.2 M). Every point is the average of three different solutions.

Calibration of methyl formate was obtained by preparation of methyl formate solutions of known concentrations in MeOH and $LiClO_4 \cdot 3H_2O$ (0.2 M). The so-prepared samples were analyzed by HPLC and their peak's area at 210 nm was integrated. Each sample was prepared three times and statistical analysis of the results afforded the calibration curve shown in **Figure S2**.

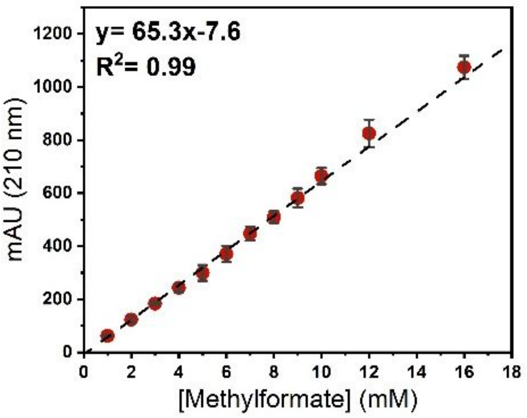

Figure S2. Calibration curve of methyl formate in MeOH ($LiClO_4$ 0.2 M). Every point is the average of three different solutions.

The faradaic efficiency (FE) for the formation of EtOAc, AcOH, or methyl formate was finally obtained according to the following equation:

$$FE = \frac{nFz}{Q} \tag{3}$$

where *n* is the amount of product formed, *F* the Faraday constant, *z* the number of electrons transferred to form one molecule of product (in this case z = 4), and *Q* the total passed charge.

Calculation of mass activity (MA), turnover numbers and frequency (TON, TOF) and of Electrochemical Active Surface Area (ECSA) for the optimized electro-adsorbed Ru_4POM@CNHs systems (protocol C)

For the calculation of the mass activity, the average current density of the 5 minutes chronoamperometries at +1.2 V vs. Ag/AgCl (J= 3.67 - 2.50 mA cm^{-2}, **Figure S36F** and Table S10) was multiplied by the electrode area (0.07 cm^2) to calculate the current value (i, mA) and normalized on the basis of the mass of the catalytically active tetraruthenate core of Ru_4POM ($[Ru_4(\mu\text{-}O)_4(\mu\text{-}OH)_2(H_2O)_4]^{6+}$ MW=574.38 g/mol) based on the Ru4POM loading (nmol/mg) on CNHs as determined by TGA analysis (**Figure S29**). The resulting MA = 2572 – 2271 A/g (eq. 4 and 5), provide a direct comparison with reported values of RuO_2-based electrocatalysts (MA RuO_2 = 34 A/g see ref: Chem. Sci., 2022, 13, 14; *Materials Today Chemistry 35 (2024) 101857,* https://doi.org/10.1016/j.mtchem.2023.101857). If we consider the MW of the overall polyoxotungstate clusters of (Ru_4POM^{10-}MW = 5459.26 g mol^{-1}) relative MAs are in the range 271- 239 A/g (see for comparison specific MA values in the range 0.5 – 850 mA/mg reported for CNHs-supported Pt/Ru nanoparticles applied to the MeOH electrooxidation reaction (MOR) in fuel cells, ref N. Tagmatarchis et al. Nanomaterials 2020, 10, 1407; doi:10.3390/nano10071407).

Mass activity for E-ads/Ru_4POM@N-CNHs:

$$\text{Mass activity}_{\text{E-ads/Ru}_4\text{POM@N-CNHs}} = \frac{0.26\ \text{mA}}{17.6\ \frac{\text{nmol}}{\text{mg}} \times 0.01\ \text{mg} \times 574.38\ \frac{\text{g}}{\text{mol}}} = 2572\ \frac{\text{A}}{\text{g}} \tag{4}$$

Mass activity for E-ads/Ru_4POM@p-CNHs:

$$\text{Mass activity}_{\text{E-ads/Ru}_4\text{POM@p-CNHs}} = \frac{0.18\ \text{mA}}{13.8\ \frac{\text{nmol}}{\text{mg}} \times 0.01\ \text{mg} \times 574.38\ \frac{\text{g}}{\text{mol}}} = 2271\ \frac{\text{A}}{\text{g}} \tag{5}$$

Calculation of the turnover number (TON)

The TON was calculated by the following equation:

$$\text{TON} = \frac{n_{\text{EtOAc}}}{n_{\text{Ru}_4\text{POM}}} \tag{6}$$

Where n_{EtOAc} refers to the amount of EtOAc (mol) detected by HPLC after 4 hours CPE and $n_{\text{Ru}_4\text{POM}}$ is the amount of Ru_4POM (nmol/mg) confined within the CNH materials, estimated from TGA analysis (**Figure S29**):

$$TON_{EtOAc} = \frac{1.49\ \frac{\text{mmol}}{\text{L}} \times 0.0025\ \text{L}}{17.6\ \frac{\text{nmol}}{\text{mg}} \times 1\ \frac{\text{mg}}{\text{mL}} \times 0.01\ \text{mL}} = 21165 \tag{7}$$

Calculation of turnover frequency (TOF)

The TOF was calculated based on the amount of EtOAc, n_{EtOAc} after 4 hours CPE, and of the amount of catalyst n_{Ru4POM}.

$$\mathrm{TOF}_{EtOAc} = \frac{n_{\mathrm{EtOAc}}}{n_{\mathrm{Ru_4POM}} \times t} = \frac{1.49\ \frac{mmol}{L} \times 0.0025\ L}{17.6\ \frac{\mathrm{nmol}}{\mathrm{mg}} \times 1\ \frac{\mathrm{mg}}{\mathrm{mL}} \times 0.01\ \mathrm{mL} \times 14400\ s} = 1.47\ \mathrm{s^{-1}} \quad (8)$$

Calculation of ECSA

The ECSA of E-ads/Ru_4POM@N-CNHs was calculated using the following equation:

$$ECSA = \frac{C_{dl}}{C_s m} = \frac{90\ \mu F}{31.1\ \mu F\ cm^{-2} \times 0.01\ mg} = 289\ cm^2/mg \qquad (9)$$

Where C_{dl} is the double layer capacitance (**Figure S37D**), C_s is the specific capacitance reported in literature for CNHs [3] and m is the overall electrocatalyst mass deposited on the electrode .

The ECSA of E-ads/Ru_4POM@p-CNHs was calculated using the following equation:

$$ECSA = \frac{C_{dl}}{C_s m} = \frac{30\ \mu F}{11.4\ \mu F\ cm^{-2} \times 0.01\ mg} = 263\ cm^2/mg \qquad (10)$$

Where C_{dl} is the double layer capacitance (**Figure S37B**), C_s is the specific capacitance reported in literature for CNHs [3] and m is the overall electrocatalyst mass deposited on the electrode.

Synthetic procedures

Ru_4POM catalyst

$Na_{10}[Ru_4(\mu\text{-}O)_4(\mu\text{-}OH)_2(H_2O)_4(\gamma\text{-}SiW_{10}O_{36})_2]$ ($Na_{10}Ru_4$POM) was synthesized according to a modified literature procedure,[7] employing an Amberlite IR-120 cationic exchange column. The column was charged with a 4 M NaCl solution, instead of the reported LiCl solution, to yield the corresponding sodium salt. The synthesis of Ru_4POM was assessed by means of FTIR spectroscopy, CV, and UV-Vis spectroscopy.

N-doped carbon nanohorns (N-CNHs)

To synthesize N-doped carbon nanohorns (N-CNHs) a slightly modified solvothermal approach was employed.[8] Pristine carbon nanohorns (p-CNHs) were ultrasonicated in water (mass concentration of p-CNHs in water: approx. 2.2 mg mL^{-1}) with hydrazine (water:hydrazine 2:1 v/v) and ammonia (water:ammonia 2:1 v/v) for 1 h at room temperature. The solution was transferred into a 40 mL Teflon lined stainless steel autoclave and heated to 150 °C for 4 h, with a muffle furnace (heating ramp: 5 °C min^{-1}). After cooling down, the N-CNHs were collected over a 0.1 µm hydrophilic PTFE filter, washed with Milli-Q® water until the filtrated reached a neutral pH. Finally, the solid was rinsed with ethanol and dried under vacuum, yielding a black powder (mass yield percentage: 86%).

Anchoring of Ru_4POM on p- and N-CNHs

For the anchoring of Ru_4POM on the p- and N-CNHs structure, the CNHs were dispersed in 0.1 M HCl (pH 1.0 – 1.2), which was prepared by diluting 37% HCl with deionized water. The mass concentration of CNHs was 10 mg L^{-1}, and the mixture was ultrasonicated for 1 h. It should be noted that the N-CNHs formed a homogeneous suspension, whereas the p-CNHs mostly floated on the surface of the 0.1 M HCl solution. $Na_{10}Ru_4$POM was then added (1.6 µmol per mg of support)[9,10] and the mixture was stirred overnight. The Ru_4POM@N-CNHs and Ru_4POM@p-CNHs were collected by vacuum filtration over a 0.1 µm hydrophilic PTFE filter, subsequently washed with 0.1 M HCl and denatured EtOH to remove unanchored Ru_4POM and chloride respectively, and finally dried. As control N-CNHs were used in this procedure without the addition of $Na_{10}Ru_4$POM, yielding H^+-N-CNHs (mass yield percentage: 98%).

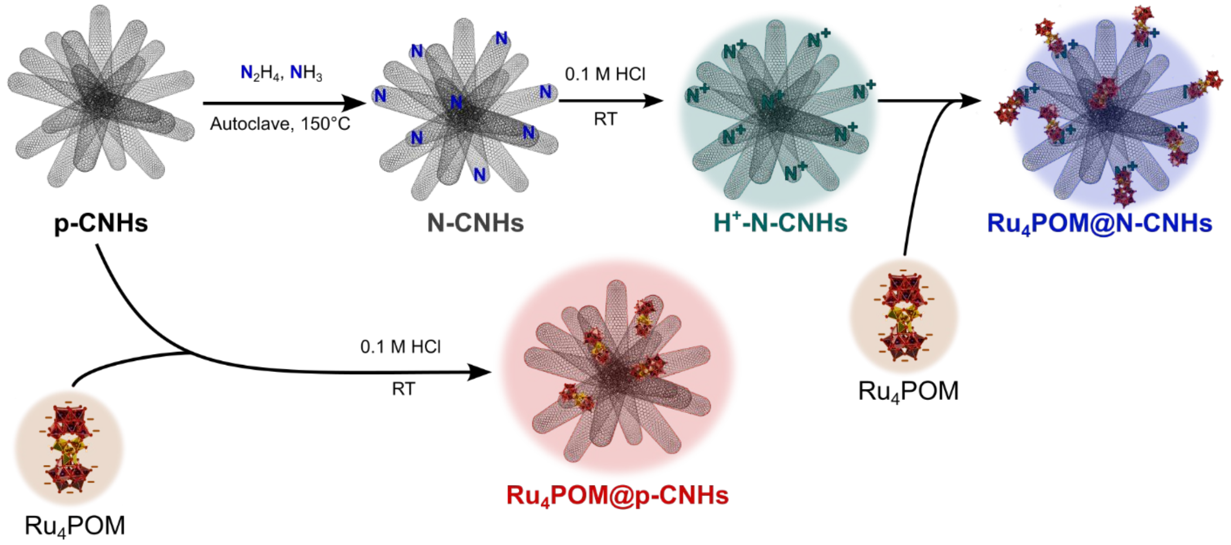

Scheme S1: Schematic illustration of the synthesis of the different tested materials.

Supporting figures

HPLC chromatographs of reference compounds

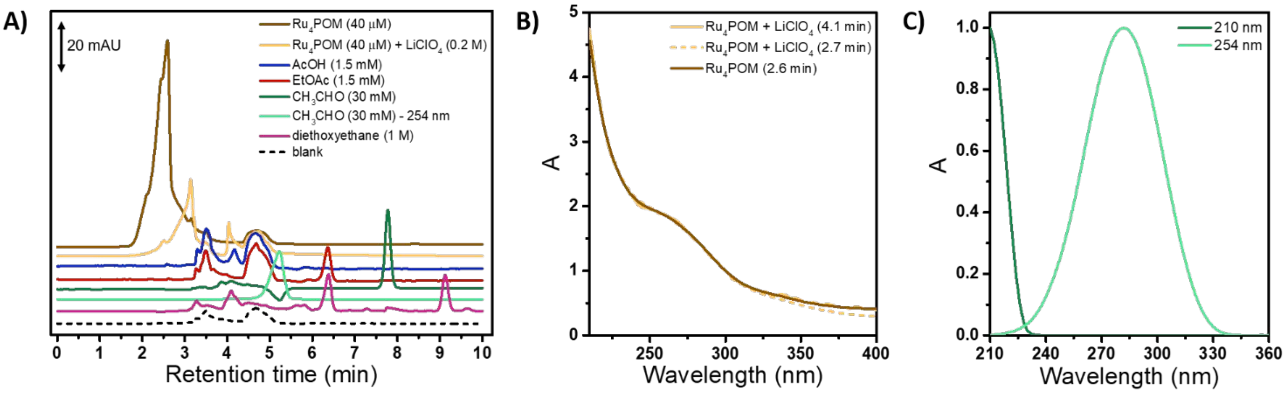

Figure S3. A. HPLC chromatograms obtained at λ_{det}= 210 nm for a blank sample (EtOH= 96%; H_2O= 4%; $LiClO_4$ 0.2 M) (black dashed line), in the presence of 1,1-diethoxyethane (violet line), in the presence of CH_3CHO (light green line at λ_{det}= 254 nm, dark green line at λ_{det}= 210 nm), in the presence of EtOAc (red line, multiplication factor: 3), in the presence of AcOH (blue line, multiplication factor: 3), in the presence of Ru_4POM (yellow line), and Ru_4POM in EtOH= 96%; H_2O= 4% without $LiClO_4$ (brown line). **B.** Normalized absorption spectra corresponding to the Ru_4POM sample (brown line, at 2.6 min) and Ru_4POM with $LiClO_4$ 0.2 M (dashed yellow line, at 2.7 min and solid yellow line, at 4.1 min). **C.** Normalized absorption spectra corresponding to the acetaldehyde sample (light green line at 254 nm, dark green line at 210 nm).

The HPLC chromatogram of a Ru_4POM solution containing $LiClO_4$ shows different peaks that have all been attributed to Ru_4POM thanks to their UV-Vis absorption spectra, which were obtained using the HPLC's diode array detector (DAD) (**Figure S3B**). Indeed, in ethanolic solutions containing $LiClO_4$, Ru_4POM can be surrounded by a different number of counterions and, in fact, it is eluted as one single peak in the absence of $LiClO_4$ (brown trace, **Figure S3A**). AcOH's peak (4.1 min) overlaps with those of Ru_4POM + $LiClO_4$, which makes the quantification of this species possible only in heterogeneous conditions. EtOAc's peak (6.3 min) does not overlap with any other. The acetaldehyde sample presents two different peaks: the first one (5.2 min) is observable at λ_{det}= 254 nm and the second one (7.7 min) is observable at λ_{det}= 210 nm. Thanks to comparison of their absorption spectra with literature reports, it was possible to attribute the first to acetaldehyde and the second one to its corresponding hemiacetal (**Figure S3C**).[11]

Homogeneous ethanol oxidation by Ru_4POM

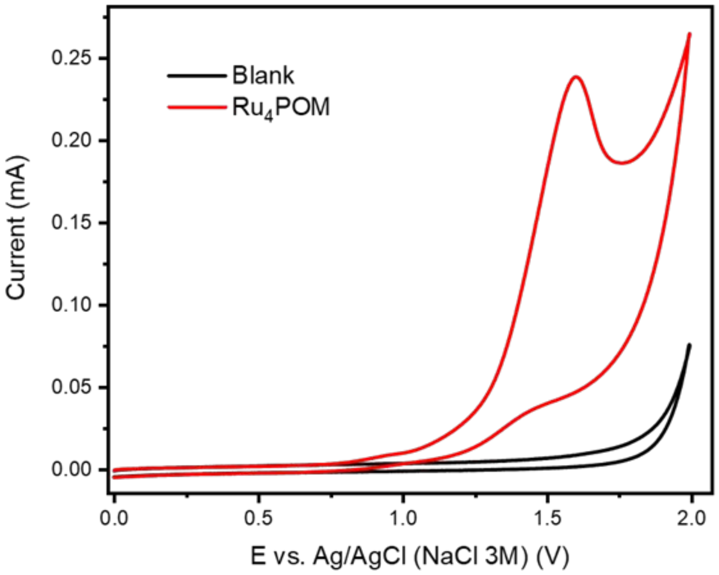

Figure S4. Cyclic voltammetry recorded for a Ru_4POM (40 µM) solution (EtOH= 96%, H_2O= 4%) (red line) and for the electrolyte (black line). WE: GC disk; RE: Ag/AgCl; CE: Pt wire; supporting electrolyte: $LiClO_4·3H_2O$ (0.2 M); υ_{scan}: 100 mV s^{-1}.

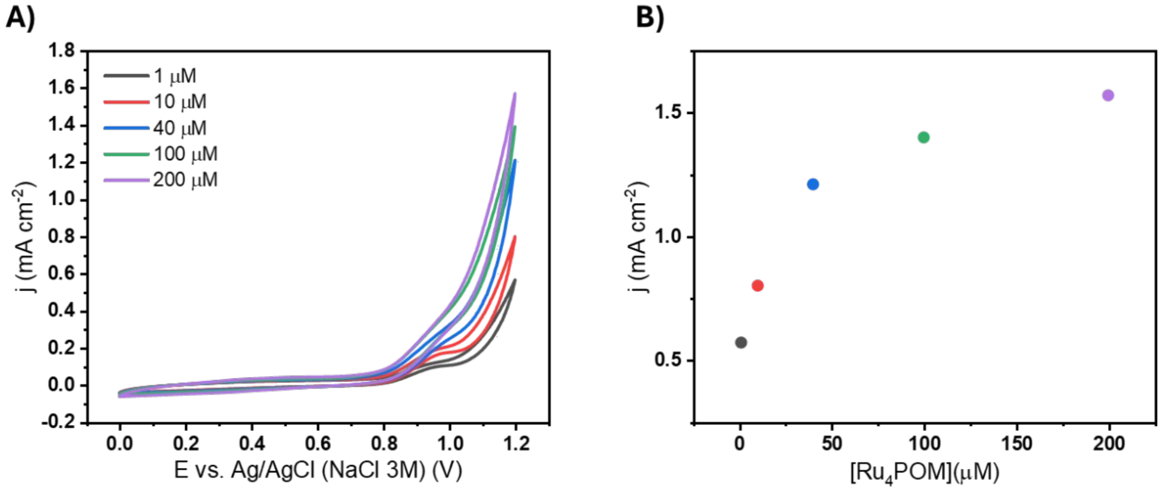

Figure S5. **A.** CVs of crescent concentration of Ru_4POM (only the 10th of ten consecutive cycles is shown); **B.** Current density at 1.2 V vs. Ag/AgCl for each concentration of Ru_4POM. WE: GC disk; RE: Ag/AgCl; CE: Pt wire; supporting electrolyte: $LiClO_4·3H_2O$ (0.2 M); υ_{scan}: 100 mV s^{-1}.

An optimal concentration of 40 µM Ru_4POM was identified, as beyond this concentration, the current density plateaued and no longer increased proportionally with the catalyst amount.

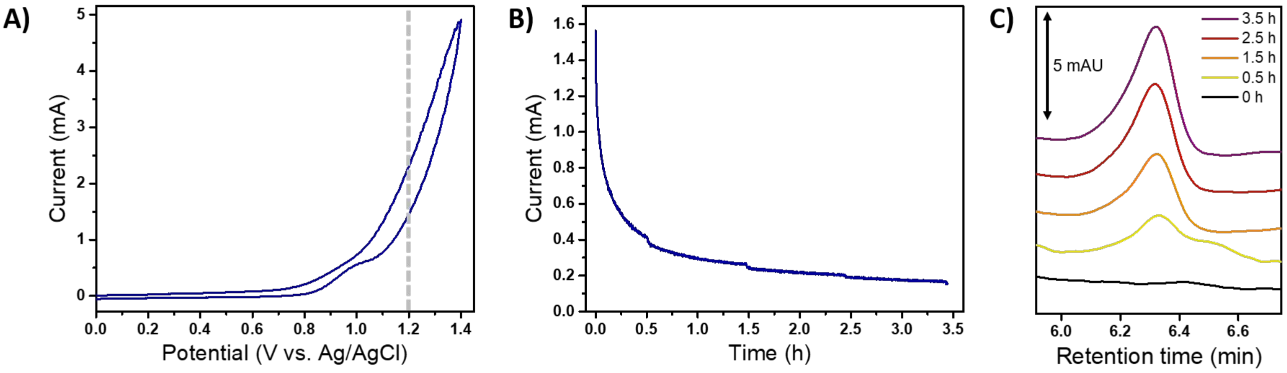

Figure S6. A. Cyclic voltammetry recorded for a Ru_4POM (40 µM) solution (EtOH= 96%, H_2O= 4%). WE: GC rod; RE: Ag/AgCl; CE: Pt wire; supporting electrolyte: $LiClO_4 \cdot 3H_2O$ (0.2 M); υ_{scan}: 100 mV s^{-1}. The vertical dashed line indicates the applied potential during the CPE. **B.** Current over time recorded applying +1.2 V vs. Ag/AgCl. **C.** HPLC peak of EtOAc detected at 210 nm and recorded over time during the CPE.

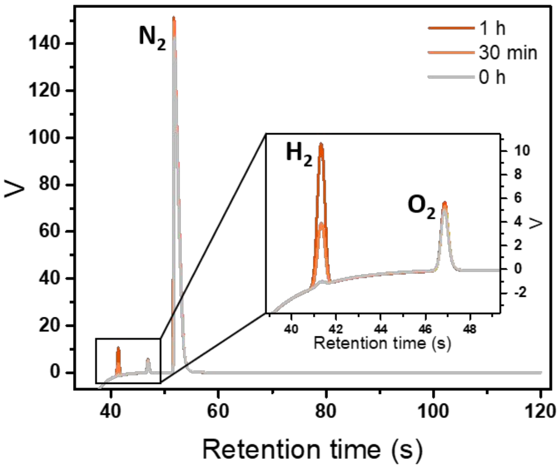

Figure S7. Gas chromatograms obtained over time via micro-GC during a CPE at +1.2 V vs. Ag/AgCl described in **Figure S6**. Electrolyte: Ru_4POM (40 µM) in EtOH= 96% (H_2O= 4%); WE: GC rod; RE: Ag/AgCl; CE: Pt wire; supporting electrolyte: $LiClO_4 \cdot 3H_2O$ (0.2 M).

Gas chromatograms show a negligible increase in O_2 concentration over the course of a 1-hour experiment, arising from minimal leaks of the sealed cell. This proves that no water oxidation reaction is taking place in these experimental conditions. The increase in H_2 concentration is attributable to the reaction taking place at the counter electrode (Pt wire). No CO peak (expected retention time: 90 s) is observed in the collected chromatograms, indicating that no overoxidation of ethanol occurs in these experimental conditions.

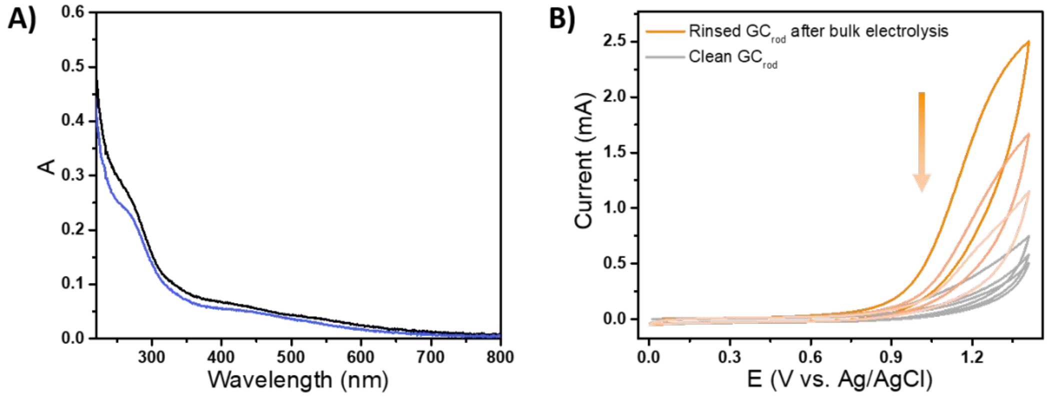

Figure S8. A. UV-Vis absorption spectra recorded before (black) and after (blue) the CPE described in **Figure S6B** upon 1:4 dilution. **B.** Consecutive cyclic voltammograms recorded in ethanol solutions (4% H_2O v/v, $LiClO_4$ 0.2 M) using a clean GC rod (gray line) and the working electrode from **Figure S6B** after CPE (orange line) and extensive rinsing with EtOH.

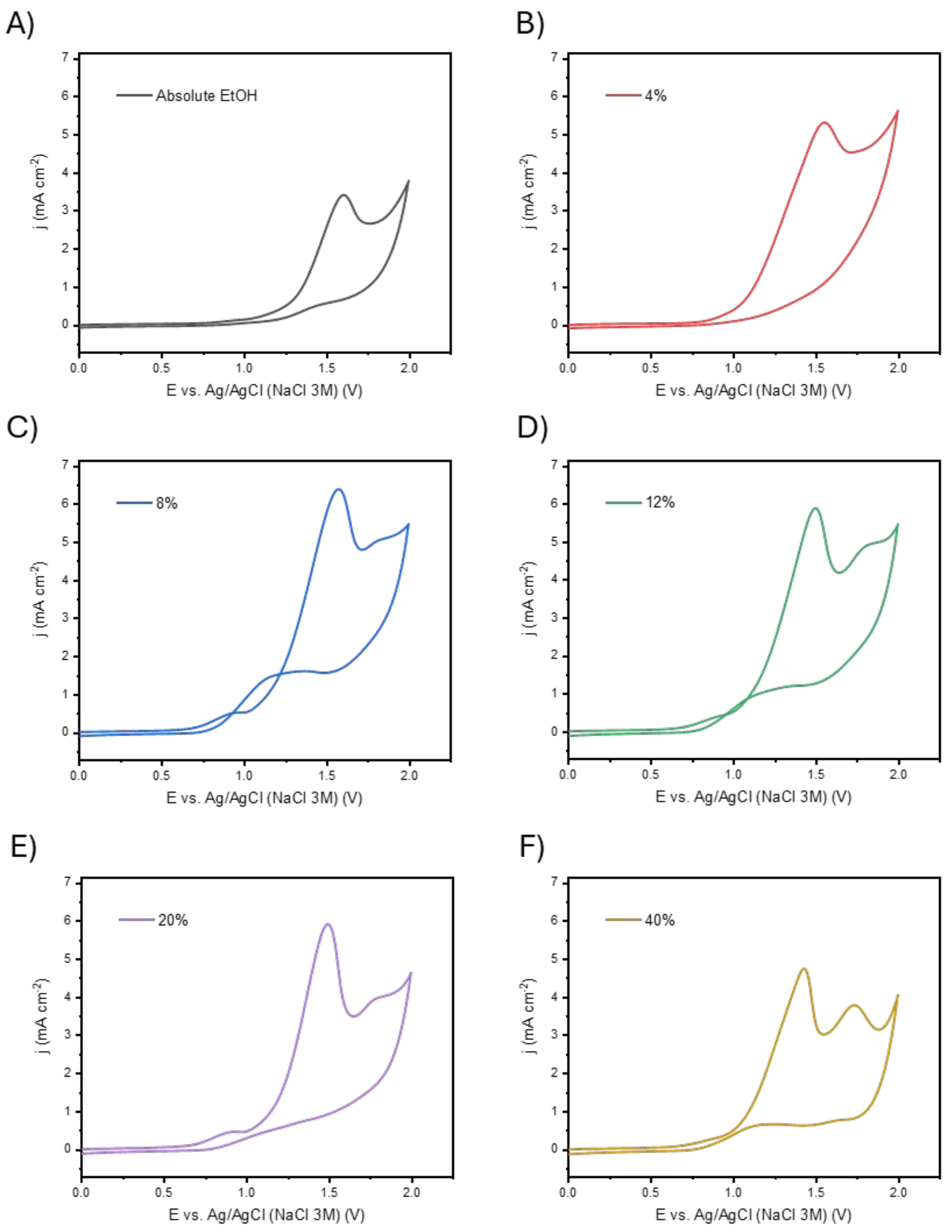

Figure S9. CV of Ru_4POM 40 µM in absolute EtOH (**A**), EtOH with 4% v/v of water (**B**), with 8% v/v of water (**C**), with 12% v/v of water (**D**), with 20% v/v of water (**E**) and with 40% of water v/v**.**

In the present study, the observed curve crossing in the cyclic voltammograms can reasonably be attributed to the Ru_4POM electrocatalyst functioning as a water oxidation catalyst (WOC), as this feature becomes evident only upon increasing the water content. Supporting this interpretation, literature reports describe a mechanism in which Ru_4POM undergoes progressive oxidation through sequential proton-coupled electron transfer (PCET) steps (electrochemical) leading to the oxygen evolution reaction (chemical), during which the reduced form of the catalyst is continuously regenerated.[11] As a consequence, if a sufficient amount of reduced catalyst accumulates at the electrode/electrolyte interface during the forward scan, it can be re-oxidized during the backward scan, thereby producing the characteristic curve crossing in the CVs."

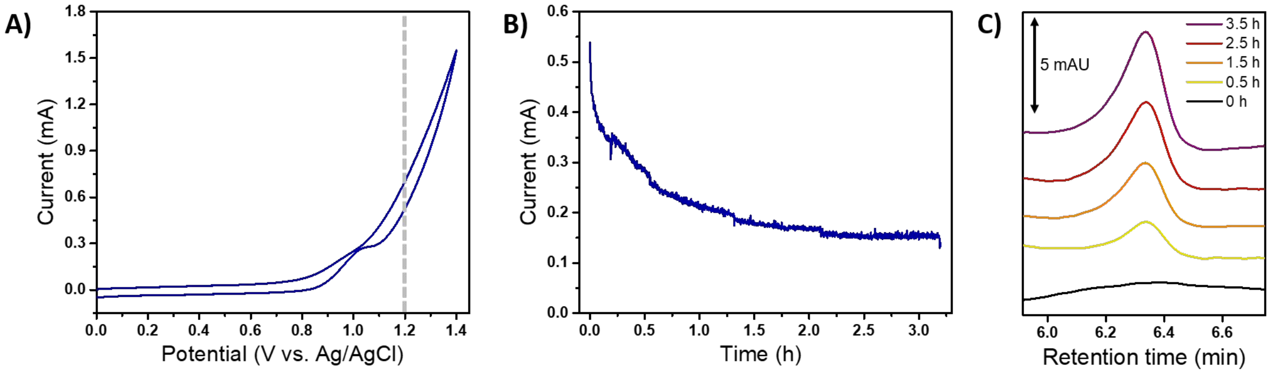

Figure S10. A. Cyclic voltammetry recorded for a Ru_4POM (40 µM) solution (EtOH= 100%). WE: GC rod; RE: Ag/AgCl; CE: Pt wire; supporting electrolyte: $LiClO_4 \cdot 3H_2O$ (0.2 M); υ_{scan}: 100 mV s^{-1}. The vertical dashed line indicates the applied potential during the CPE. **B.** Current over time recorded applying +1.2 V vs. Ag/AgCl. **C.** HPLC peak of EtOAc detected at 210 nm and recorded over time during the CPE.

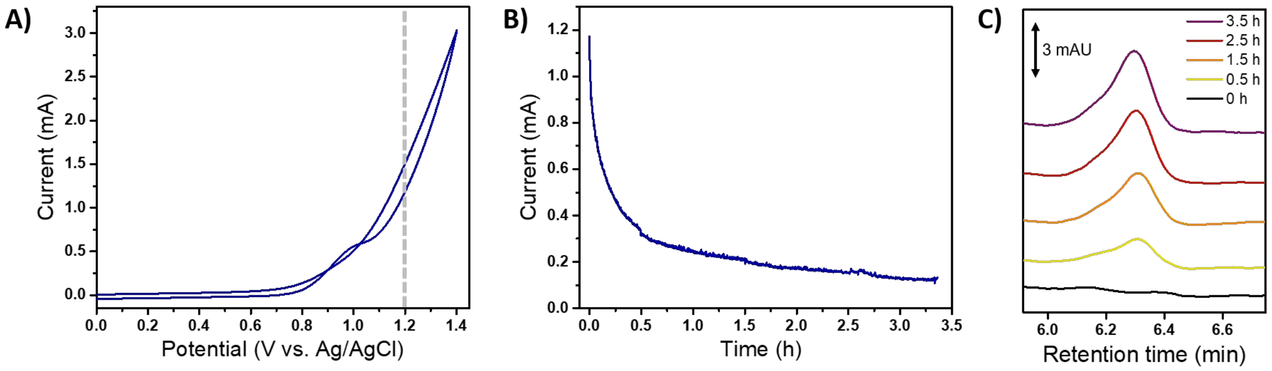

Figure S11. A. Cyclic voltammetry recorded for a Ru_4POM (40 µM) solution (EtOH= 92%, H_2O= 8%). WE: GC rod; RE: Ag/AgCl; CE: Pt wire; supporting electrolyte: $LiClO_4 \cdot 3H_2O$ (0.2 M); υ_{scan}: 100 mV s^{-1}. The vertical dashed line indicates the applied potential during the CPE. **B.** Current over time recorded applying +1.2 V vs. Ag/AgCl. **C.** HPLC peak of EtOAc detected at 210 nm and recorded over time during the CPE.

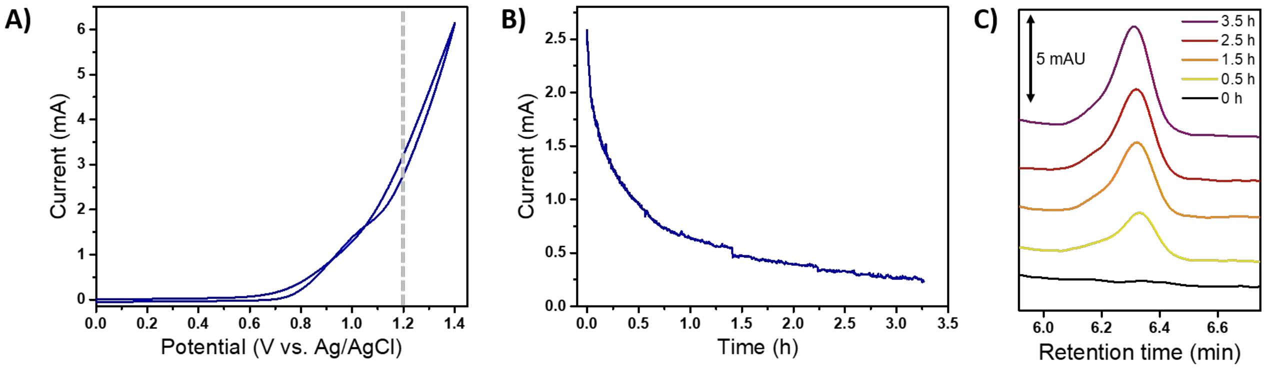

Figure S12. A. Cyclic voltammetry recorded for a Ru_4POM (40 µM) solution (EtOH= 80%, H_2O= 20%). WE: GC rod; RE: Ag/AgCl; CE: Pt wire; supporting electrolyte: $LiClO_4 \cdot 3H_2O$ (0.2 M); υ_{scan}: 100 mV s^{-1}. The vertical dashed line indicates the applied potential during the CPE. **B.** Current over time recorded applying +1.2 V vs. Ag/AgCl. **C.** HPLC peak of EtOAc detected at 210 nm and recorded over time during the CPE.

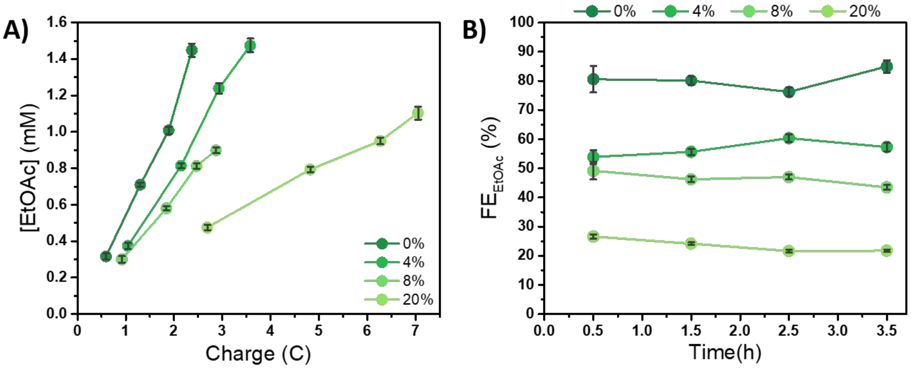

Figure S13. A. EtOAc concentration vs. passed charge during CPEs shown in **Figure S6, S10-S12**. **B.** FE_{EtOAc} vs. time during CPEs shown in **Figure S6, S10-S12**.

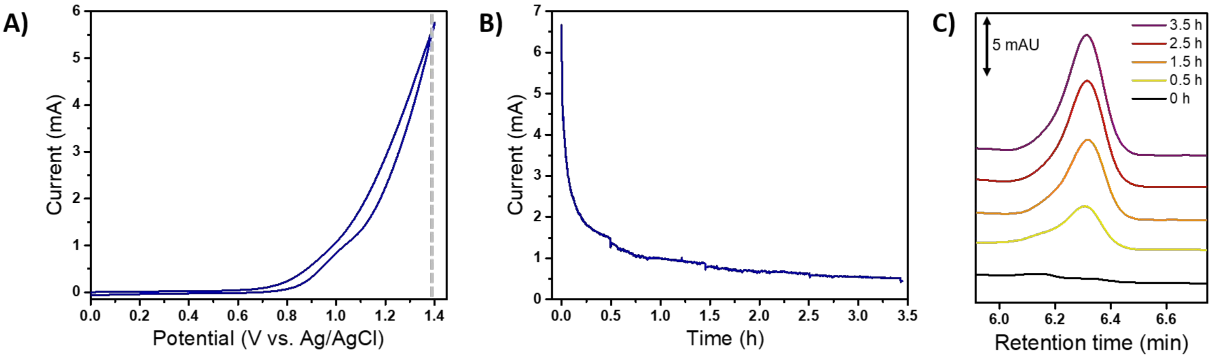

Figure S14. A. Cyclic voltammetry recorded for a Ru_4POM (40 µM) solution (EtOH= 96%, H_2O= 4%). WE: GC rod; RE: Ag/AgCl; CE: Pt wire; supporting electrolyte: $LiClO_4 \cdot 3H_2O$ (0.2 M); υ_{scan}: 100 mV s^{-1}. The vertical dashed line indicates the applied potential during the CPE. **B.** Current over time recorded applying +1.4 V vs. Ag/AgCl. **C.** HPLC peak of EtOAc detected at 210 nm and recorded over time during the CPE.

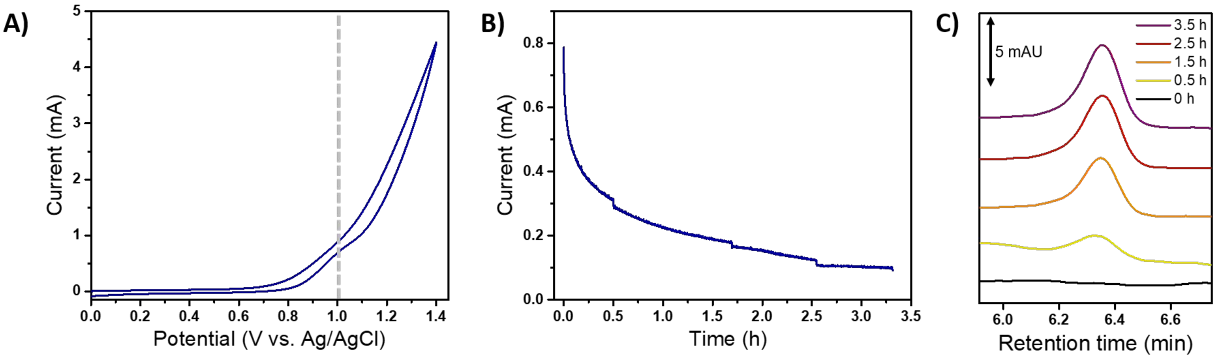

Figure S15. A. Cyclic voltammetry recorded for a Ru_4POM (40 µM) solution (EtOH= 96%, H_2O= 4%). WE: GC rod; RE: Ag/AgCl; CE: Pt wire; supporting electrolyte: $LiClO_4 \cdot 3H_2O$ (0.2 M); υ_{scan}: 100 mV s^{-1}. The vertical dashed line indicates the applied potential during the CPE. **B.** Current over time recorded applying +1.0 V vs. Ag/AgCl. **C.** HPLC peak of EtOAc detected at 210 nm and recorded over time during the CPE.

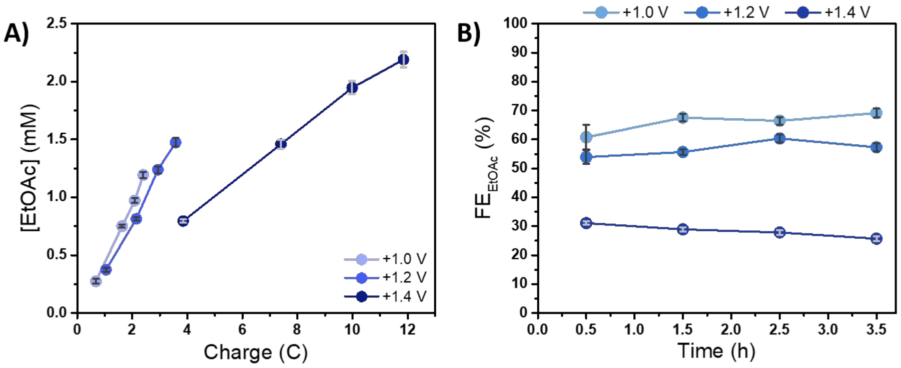

Figure S6. A. EtOAc concentration vs. passed charge during CPEs shown in **Figure S6, S14** and **S15**. **B.** FE_{EtOAc} vs. time during CPEs shown in **Figure S6, S14** and **S15**.

Table S1 EtOAc analytical yields obtained with CPE experiments (Protocol A) under different experimental conditions. Ru_4POM (40 µM) in EtOH= 100-x% (H_2O= x%); WE: GC rod; RE: Ag/AgCl; CE: Pt wire; supporting electrolyte: $LiClO_4 \cdot 3H_2O$ (0.2 M).

Water content (%)	Applied potential (V vs. Ag/AgCl)	EtOAc yield (µmol)
0	+1.2	3.60
4	+1.2	3.67
8	+1.2	2.22
20	+1.2	2.70
4	+1.0	2.97
4	+1.4	5.47

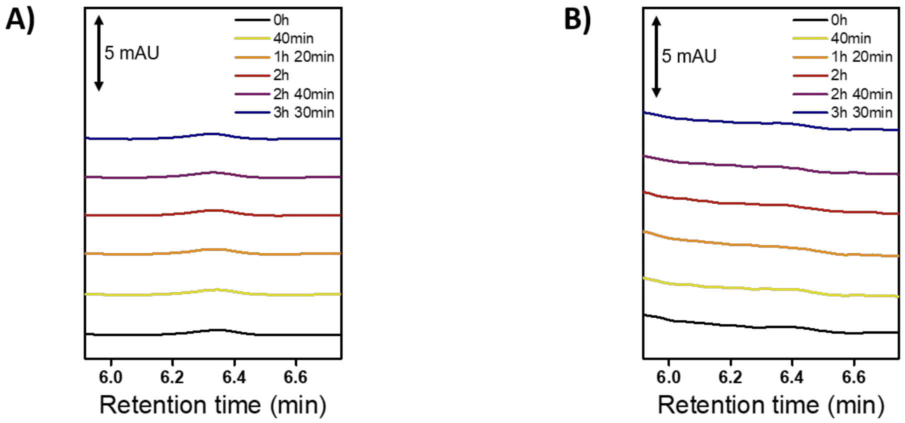

Figure S17. A. HPLC chromatographs detected at 210 nm showing no formation of EtOAc over time in an ethanol solution (EtOH= 96%, H_2O= 4%) containing Ru_4POM (40 µM), acetic acid (27 mM) and $LiClO_4 \cdot 3H_2O$ (0.2 M). **B.** HPLC chromatographs detected at 210 nm showing no formation of EtOAc over time in an ethanol solution (EtOH= 96%, H_2O= 4%) containing Ru_4POM (40 µM) and $LiClO_4 \cdot 3H_2O$ (0.2 M), with no applied bias.

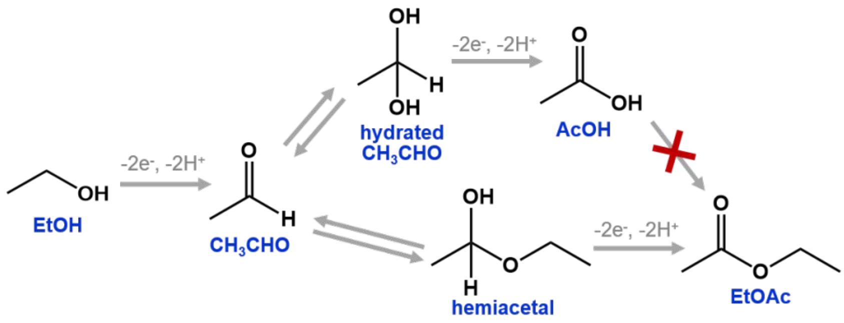

Figure S18. Proposed mechanism for the ethanol oxidation reaction catalyzed by Ru_4POM.

After initial bielectronic oxidation of EtOH to acetaldehyde, two possible equilibria exist: i) hydration by addition of an H_2O molecule; ii) hemiacetal formation by addition of an EtOH molecule. A further two-electron oxidation of these intermediates finally leads to AcOH and EtOAc formation, respectively. Since in our standard reaction conditions (wet ethanol, i.e. 4% v/v H_2O) equilibrium with EtOH is strongly shifted towards hemiacetal formation, the dominant observed product is EtOAc. The proposed mechanism agrees with the results reported by Liu *et. al*,[12] who did not report EtOAc formation. Indeed, hemiacetal formation is strongly disfavored in their harsher (EtOH with 0.5 M H_2SO_4) or more diluted (pH 7.0 buffer solution with 1.0 M EtOH) reaction conditions, leading to AcOH being the only observable 4-electron oxidation product. In this system, the formation of acetaldehyde and AcOH is ≥ 95.9%, 1,1-diethoxyethane and EtOAc are present only in trace amounts.

Homogeneous methanol oxidation by Ru_4POM

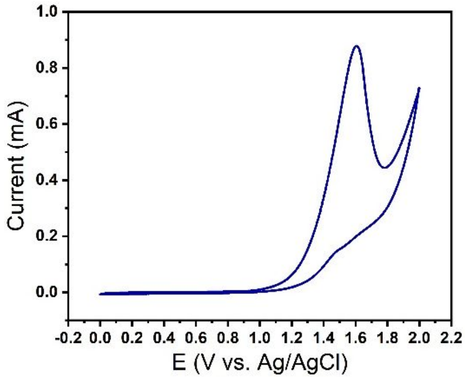

Figure S19. Cyclic voltammetry recorded for a Ru_4POM (40 µM) solution (MeOH 100%). WE: GC disk; RE: Ag/AgCl; CE: Pt wire; supporting electrolyte: $LiClO_4{\cdot}3H_2O$ (0.2 M); υ_{scan}: 100 mV s^{-1}.

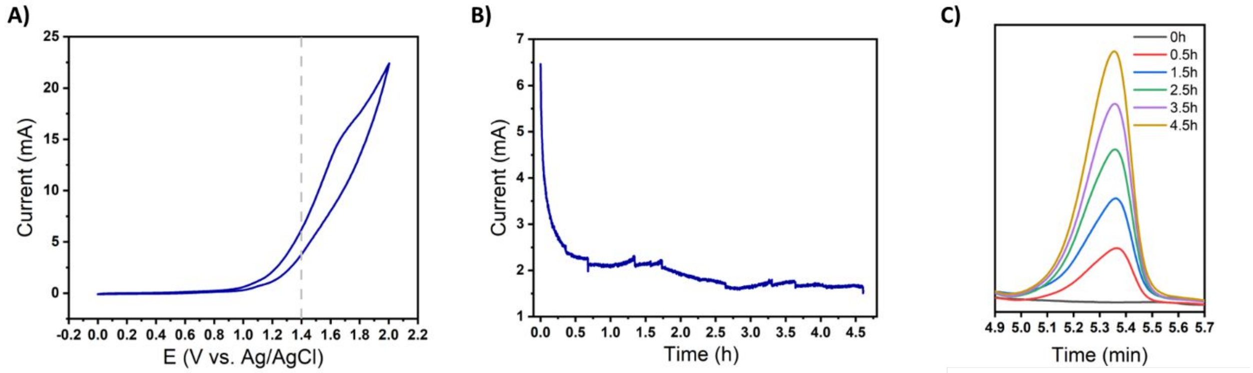

Figure S20. A. Cyclic voltammetry recorded for a Ru_4POM (40 µM) solution (MeOH 100%). WE: GC rod; RE: Ag/AgCl; CE: Pt wire; supporting electrolyte: $LiClO_4{\cdot}3H_2O$ (0.2 M); υ_{scan}: 100 mV s^{-1}. The vertical dashed line indicates the applied potential during the CPE. **B.** Current over time recorded applying +1.4 V vs. Ag/AgCl. **C.** HPLC peak of methyl formate detected at 210 nm and recorded over time during the CPE.

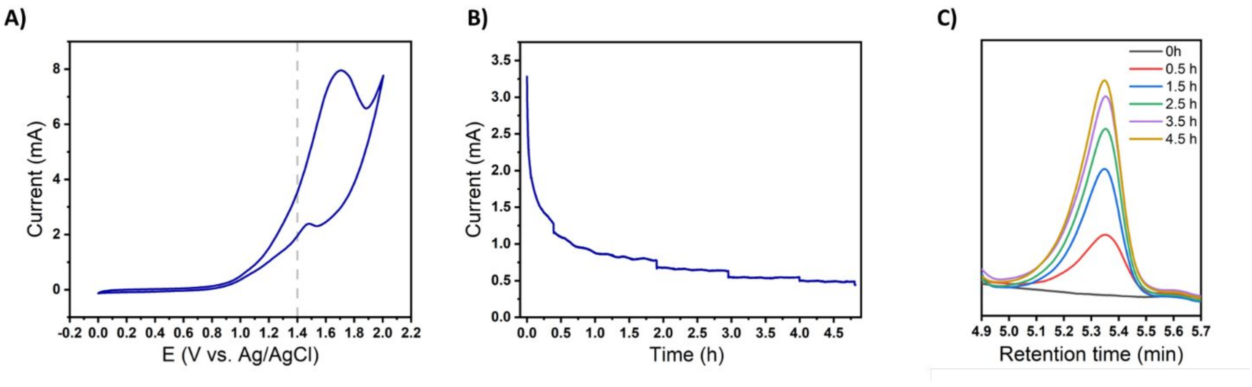

Figure S21. A. Cyclic voltammetry recorded for a Ru_4POM (40 µM) solution (MeOH 96%, water 4%). WE: GC rod; RE: Ag/AgCl; CE: Pt wire; supporting electrolyte: $LiClO_4{\cdot}3H_2O$ (0.2 M); υ_{scan}: 100 mV s^{-1}. The vertical dashed line indicates the applied potential

during the CPE. **B.** Current over time recorded applying +1.4 V vs. Ag/AgCl. **C.** HPLC peak of methyl formate detected at 210 nm and recorded over time during the CPE.

Given the commercial interest in milder, selective methyl formate synthesis,[13–15] Ru_4POM was tested for methanol oxidation, as well. In this case, the catalytic wave has a slightly higher onset potential (+1.15 V vs. Ag/AgCl, **Figure S19**) and therefore CPEs were performed at +1.4 V vs. Ag/AgCl for up to 4.5 h. In these conditions, a FE for methyl formate formation (FE_{MF}) of 79±5% was measured by HPLC, and no other products were detected with the same instrumentation (**Figure S20**). The average FE_{MF} detected for a CPE experiment over 4.5 h performed at +1.4 V vs. Ag/AgCl in the presence of 4% v/v of water is 86±9% (**Figure S21**). This value is higher compared to the one obtained for EtOAc formation in comparable conditions, in agreement with the higher equilibrium constant ratio for hemiacetal formation (K_{hemi}) or hydration (K_{hydr}) reported for formaldehyde in MeOH (K_{hemi}/K_{hydr}= 0.575) compared to acetaldehyde in EtOH (K_{hemi}/K_{hydr}= 0.472).[16] This further supports the proposed mechanism shown in **Figure 2 and S18**. Moreover, in the corresponding CV, a quasi-reversible wave appears at +1.7 V (ΔV= 160 mV) which could be due to a ruthenium redox process. In contrast to what observed for ethanol, the addition of water caused a consistent decrease in the measured current but did not seem to have a relevant role in the reaction selectivity.

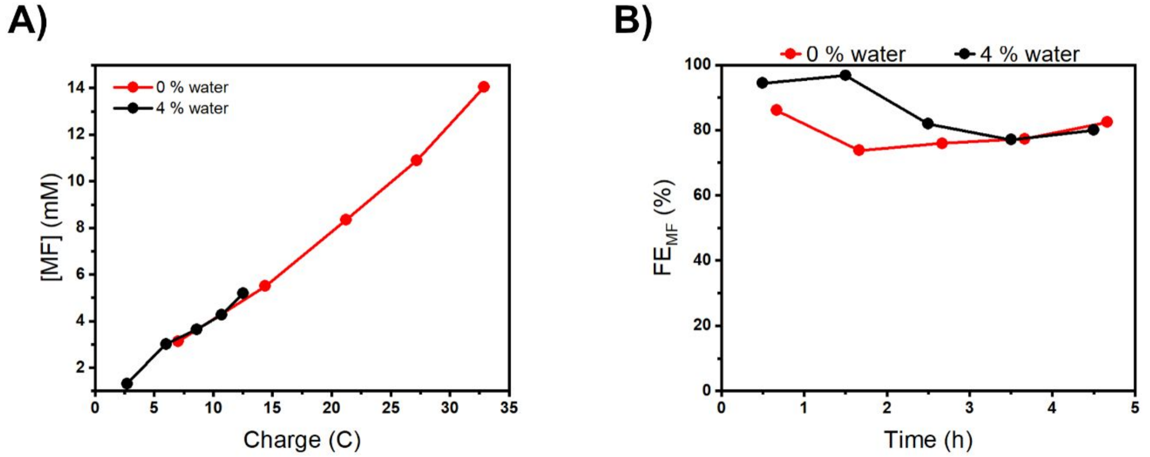

Figure S22. A. Methyl formate concentration vs. passed charge during CPEs shown in **Figure S20** (red) and **Figure S21** (black). **B.** FE_{MF} vs. time for the CPEs shown in **Figure S20** (red) and **Figure S21** (black).

XPS characterization of CNHs and $Ru_4POM@CNHs$ hybrid materials

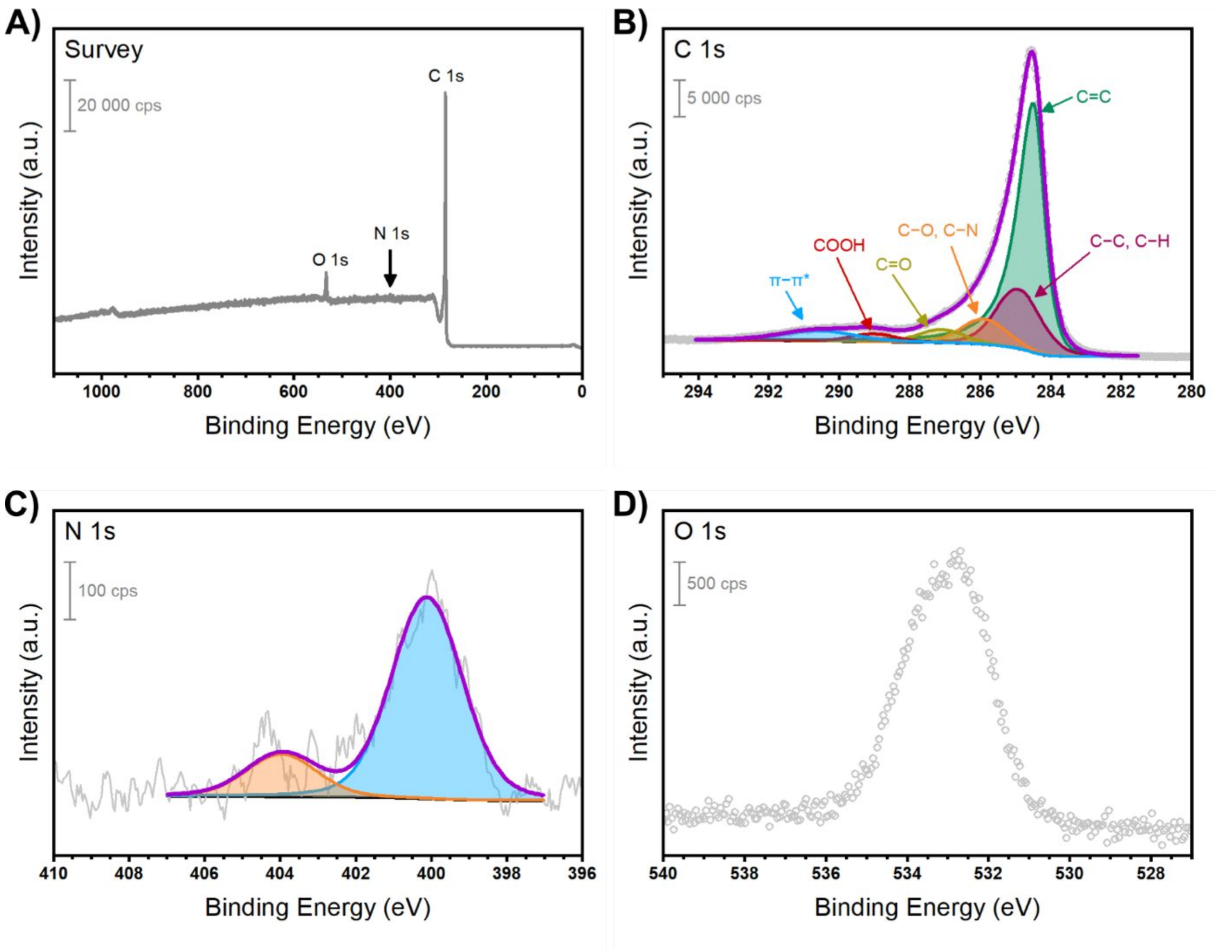

Figure S23. XPS analysis of N-CNHs. Experimental data in grey lines or circles and curve fitting in colored lines. The black line in **B** and **C** represents the background and the purple line the envelope of the curve fitting. **A.** Survey spectrum; **B.** high-resolution C 1s spectrum; **C)** high-resolution N 1s spectrum; **D.** high-resolution O 1s spectrum.

In the survey no elements other than C, O, and N were found. Based on the high-resolution spectra an atomic concentration of 95.30% C, 3.97% O, and 0.72% N was calculated. The N 1s spectrum in **Figure S23C** was curve fitted to two contributions, attributed to backbone inserted N (blue curve) and oxidized N (orange curve).[17–21]

Table S2. XPS results of N-CNHs.

	Position (eV)	FWHM (eV)	Reference
**C=C**	284.5	0.7	[22]
**C−C, C−H**	284.9	1.5	[22]
**C−O, C−N**	285.9	1.5	[22,23]*
**C=O**	287.1	1.5	[22]*
**COOH**	289.0	1.5	[22]
**π -π***	290.6	2.5	[22]*
**Backbone inserted N**	400.3	2.2	[17–20]
**Oxidized N**	404.2	2.2	[17–21]

*small differences to literature might be due to different line shapes or undetected contributions. However, since the main focus was not on the C 1s, this issue was not further investigated.

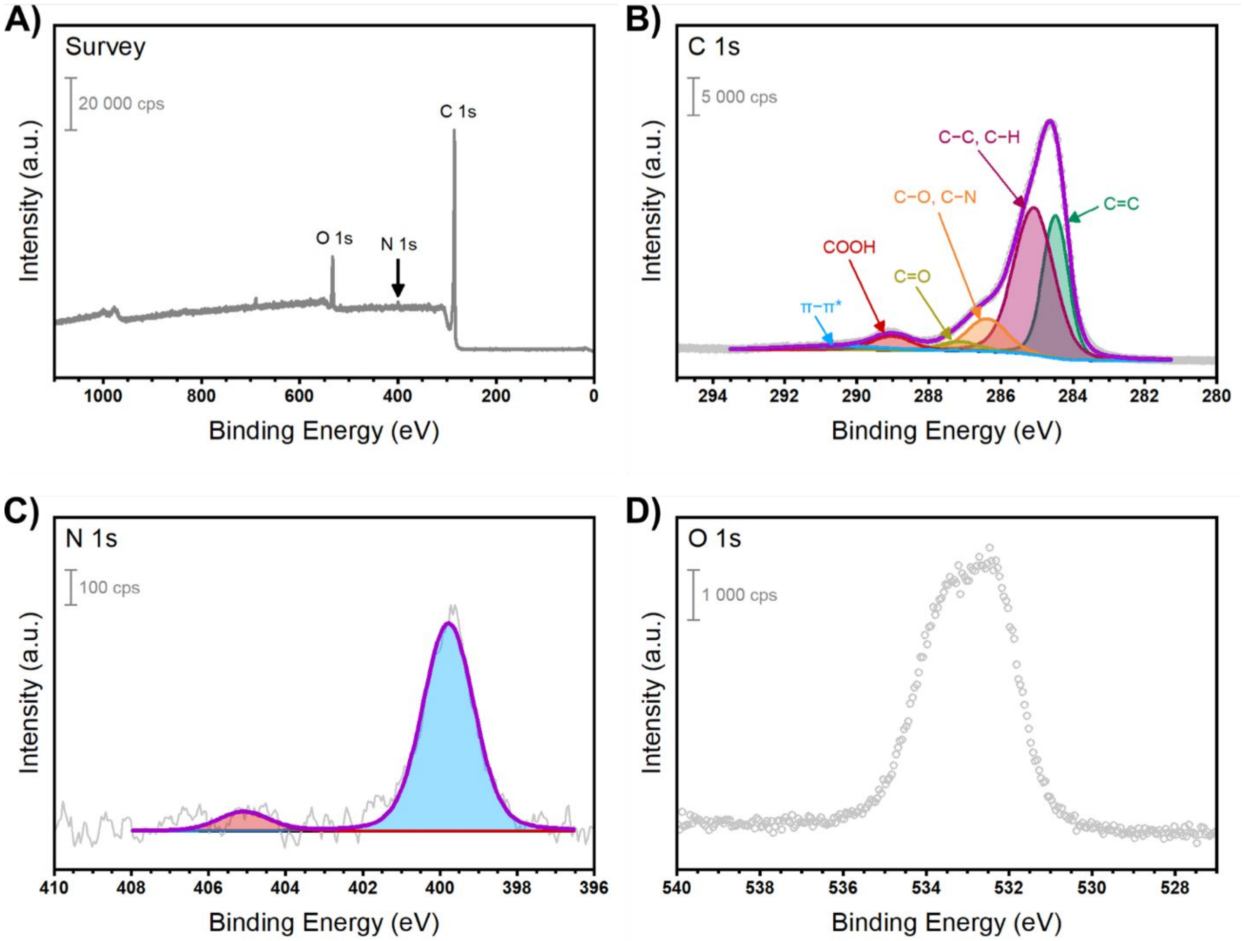

Figure S24. XPS analysis of H^+-N-CNHs. Experimental data in grey lines or circles and curve fitting in colored lines. The black line in **B** and **C** represents the background and the purple line the envelope of the curve fitting. **A.** Survey spectrum; **B.** high-resolution C 1s spectrum; **C.** high-resolution N 1s spectrum; **D.** high-resolution O 1s spectrum.

The N 1s spectrum in **Figure S24C** was curve-fit to two contributions, attributed to backbone inserted N (blue curve) and proposed protonated N (red curve). However, the presence of the latter fitting could be ignored due to its very low intensity (c.f. Figure S24 E)

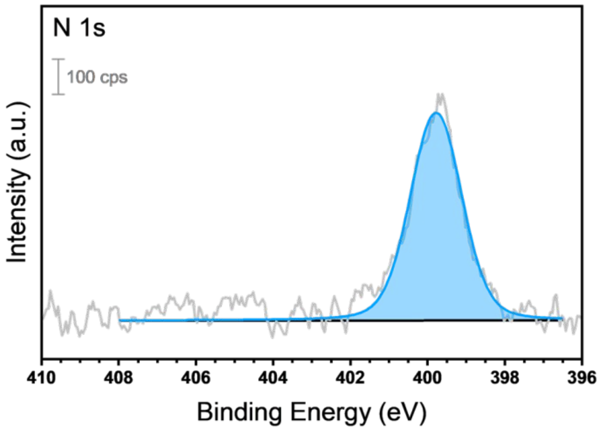

Figure S24E. XPS analysis of H^+-N-CNHs. Experimental data in grey lines or circles and curve fitting in colored lines. The blue line refers to the high-resolution N 1s spectrum.

Table S3. XPS results of H^+-N-CNHs.

	Position (eV)	FWHM (eV)	Reference
**C=C**	284.5	0.8	[22]
**C−C, C−H**	285.1	1.3	[22]*
**C−O, C−N**	286.4	1.3	[22]
**C=O**	287.2	1.3	[22]*
**COOH**	289.0	1.3	[22]
**π -π***	290.5	3.0	[22]*
**Backbone inserted N**	399.8	1.6	[17–20]
**Oxidized N**	405.1	1.6	[17–20]

*small differences to literature might be due to different line shapes or undetected contributions. However, since the main focus was not on the C 1s, this issue was not further investigated.

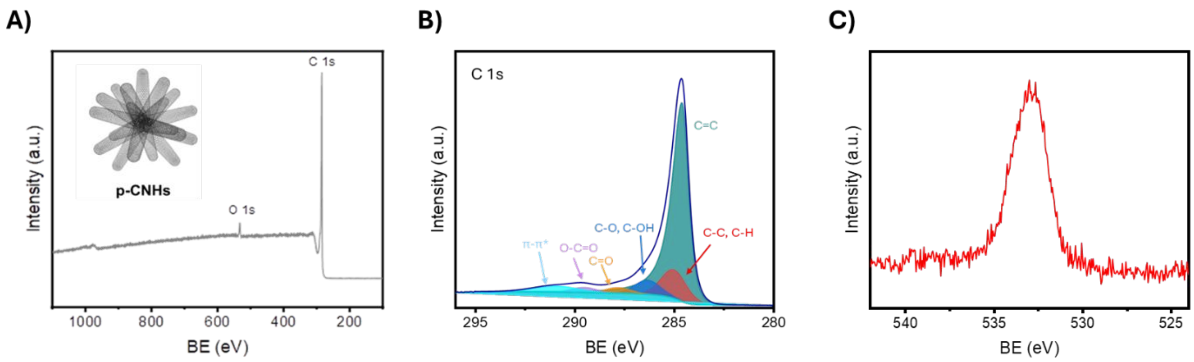

Figure S7. A. Survey spectrum of p-CNHs **B.** high resolution C 1s spectrum; **C.** high-resolution O 1s spectrum.

Only carbon and oxygen signals are visible by XPS analysis of p-CNHs, as expected. The high-resolution C 1s spectrum (**Figure S25B**) shows a less defective material than the nitrogen doped one, as seen comparing the Raman spectra (**Figure S30**).

Table S4. XPS results of p-CNHs.

	Position (eV)	FWHM (eV)	Reference
**C=C**	284.5	1.1	[22]
**C−C, C−H**	285.1	1.4	[22*]
**C−O, C−OH**	286.3	1.8	[22]
**C=O**	287.9	7.1	[22*]
**COOH**	289.5	7.4	[22]
**π -π***	291.1	7.6	[22*]
**O 1s**	533	2.4	

*small differences to literature might be due to different line shapes or undetected contributions. However, since the main focus was not on the C 1s, this issue was not further investigated.

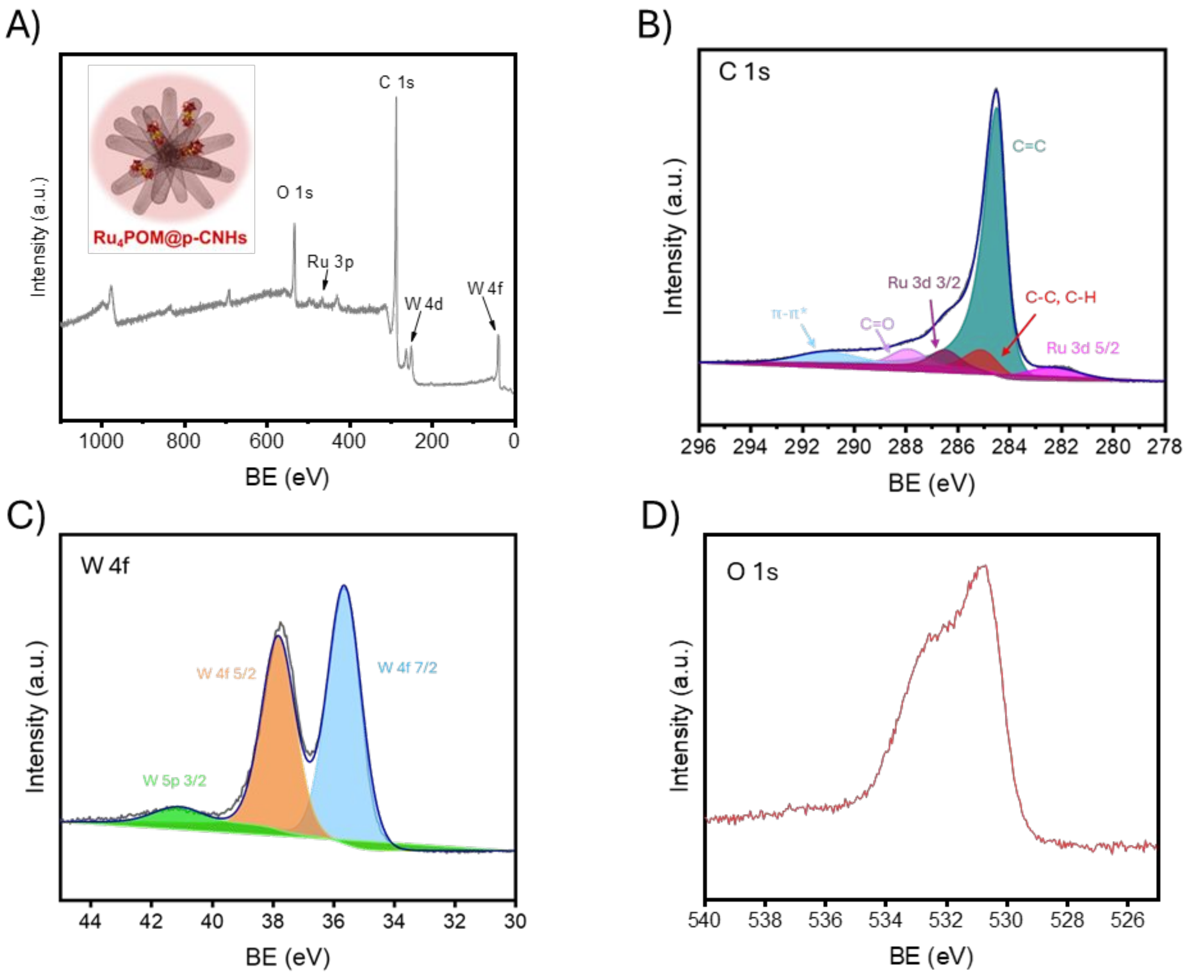

Figure S26. XPS analysis of Ru_4POM@p-CNHs. Experimental data in grey lines and curve fitting in colored lines. The black line in **B** and **C** represents the background and the blue line the envelope of the curve fitting. **A.** Survey spectrum; **B.** high-resolution C 1s spectrum; **C.** high-resolution W 4f spectrum; **D.** high-resolution O 1s spectrum.

Table S5. XPS results of Ru_4POM@p-CNHs.

	**Position (eV)**	**FWHM (eV)**	**Reference**
**Ru 3d 5/2**	282.3	2.2	[24]
**C=C**	284.5	1.1	[22]
**C−C, C−H**	285	1.3	[22*]
**Ru 3d 3/2**	286.5	2.4	[24]
**C=O**	287.9	3.5	[22]
**π -π***	291.1	6.1	[22*]
**Metal−O**	530.3	3.3	[25]
**W 4f 7/2**	35.6	1.3	[9,25]
**W 4f 5/2**	37.8	1.4	[9,25]
**W 5p 3/2**	41	3.6	

*small differences to literature might be due to different line shapes or undetected contributions. However, since the main focus was not on the C 1s, this issue was not further investigated.

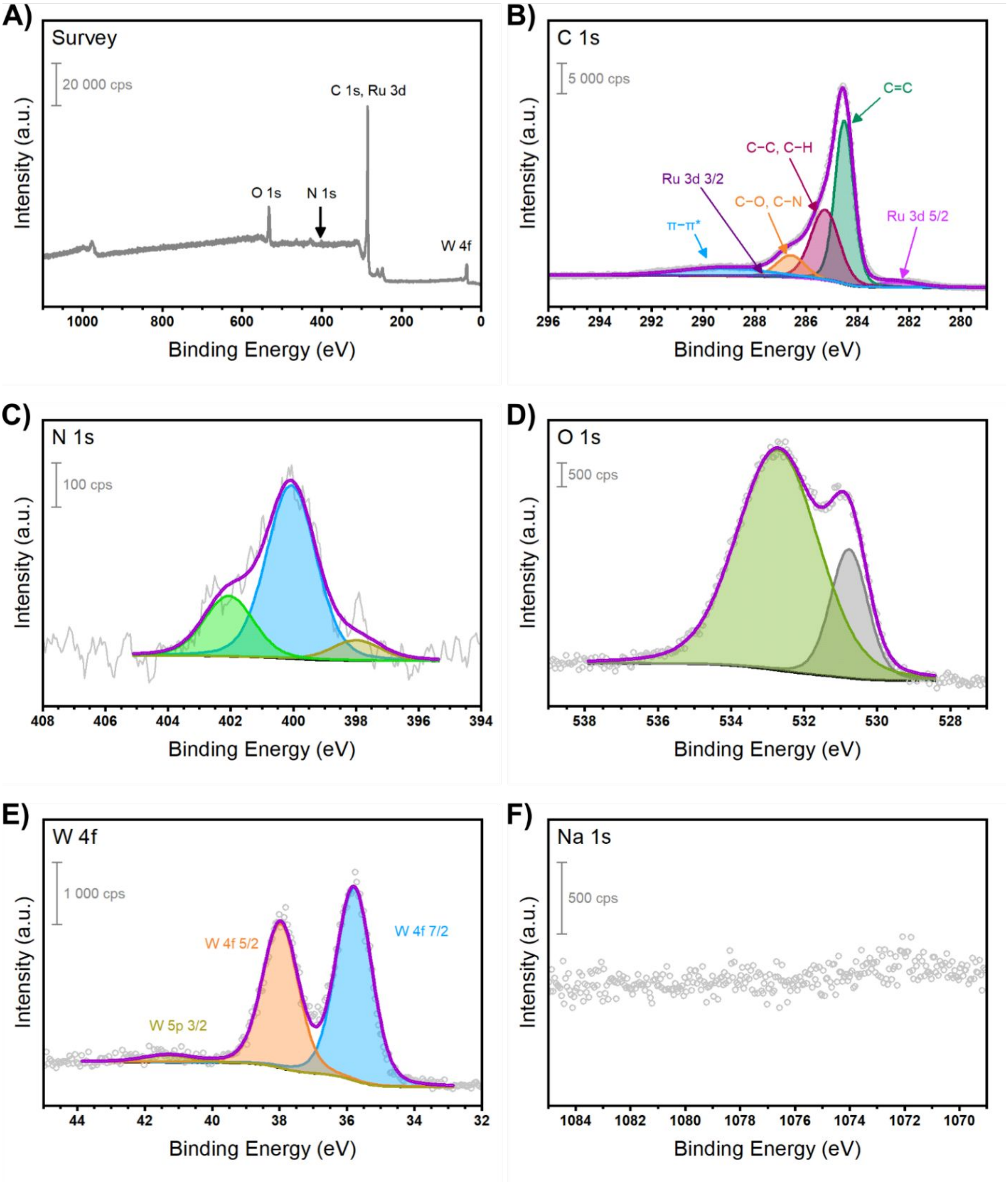

Figure S27. XPS analysis of Ru_4POM@N-CNHs. Experimental data in grey lines or circles and curve fitting in colored lines. The black line in **B**–**E** represents the background and the purple line the envelope of the curve fitting. **A.** Survey spectrum; **B.** high-resolution C 1s spectrum; **C.** high-resolution N 1s spectrum; **D.** high-resolution O 1s spectrum; **E.** high-resolution W 4f spectrum; **F.** high-resolution Na 1s spectrum.

In the survey spectrum of Ru_4POM@p-CNHs and Ru_4POM@N-CNHs in **Figure S26A and S27A** W was found, suggesting the presence of the Ru_4POM catalyst. The high-resolution C 1s spectrum in **Figure S26B and S27B** also showed a shoulder at 282.5 eV binding energy, which can be rationalized with the presence of Ru 3d 5/2. The high-resolution N 1s spectrum in **Figure S27C** showed the emergence of other contributions besides the previously observed backbone inserted N (blue curve). The green curve might be attributed to protonated N with anchored Ru_4POM, while the yellow curve might be due to non-protonated N with the anchored Ru_4POM in close proximity. The O 1s high-resolution spectrum in **Figure S26D and S27D** also shows the emergence of a new peak at 530.8 eV binding energy, which is attributed to the metal−O bond of the Ru_4POM (grey curve). The olive-colored curve illustrates the envelope of

contributions of the support as observed in the previous samples. The high-resolution spectrum of W 4f in **Figure S26C and S27E** corroborates the existence of Ru_4POM on the samples. No Na was found in the high-resolution spectrum of Na 1s in **Figure S27F**.

Table S6. XPS results of Ru_4POM@N-CNHs.

	Position (eV)	FWHM (eV)	Reference
**Ru 3d 5/2**	282.5	2.0	[24]
**C=C**	284.5	0.9	[22]
**C−C, C−H**	285.3	1.3	[22*]
**C−O, C−N**	286.6	1.3	[22]
**Ru 3d 3/2**	286.7	5	[24]
**π -π***	289.1	4.2	[22*]
**Non-protonated N with Ru_4POM close by (suggestion)**	398.0	1.9	
**Backbone inserted N**	400.1	1.9	[17–20]
**Protonated N with Ru_4POM close by (suggestion)**	402.1	1.9	
**Metal−O**	530.8	1.2	[25]
**W 4f 7/2**	35.8	1.2	[9,25]
**W 4f 5/2**	38.0	1.2	[9,25]
**W 5p 3/2**	41.3	1.7	

*small differences to literature might be due to different line shapes or undetected contributions. However, since the main focus was not on the C 1s, this issue was not further investigated.

Transmission electron microscopy (TEM), thermogravimetric analysis (TGA), Raman spectroscopy and Zeta potential of CNHs and Ru_4POM@CNHs hybrid materials

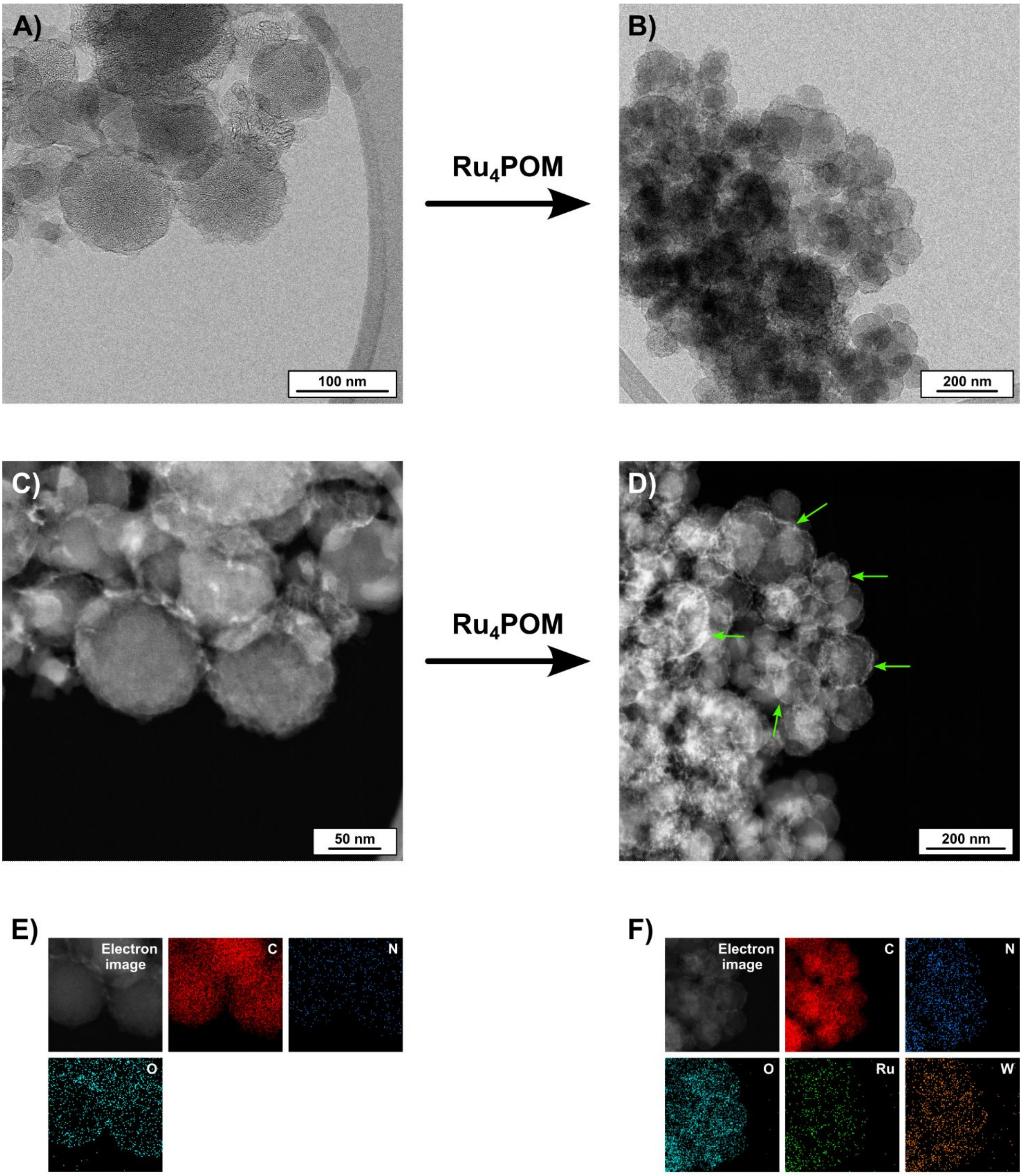

Figure S28. TEM analysis probing the success of anchoring Ru_4POM on the N-CNHs support. **A**, **B.** TEM bright field (BF) images of N-CNHs and Ru_4POM@N-CNHs, respectively. **C**, **D.** High-angle annular dark-field (HAADF) imaging of the area shown in **A** and **B**, respectively. The green arrows indicate areas with higher Z-contrast on the rim of the N-CNHs **E** and **F** Energy-dispersive X-ray spectroscopy (EDX) on an area shown in **C** and **D**, respectively (square size for EDX images: 200 nm x 200 nm for **E** and 500 nm x 500 nm for **F**).

TEM analysis confirmed an unchanged shape upon Ru_4POM anchoring on the N-CNHs support. The appearance of multiple bright spots on the rim of the CNHs in **Figure S28D**, highlighted with green arrows, due to Z-contrast can be reasoned with the presence of Ru_4POM, as further corroborated by EDX mapping in **Figure S28F** with the appearance of Ru and W. In addition, the elements have been shown to be homogeneously dispersed on the CNHs.

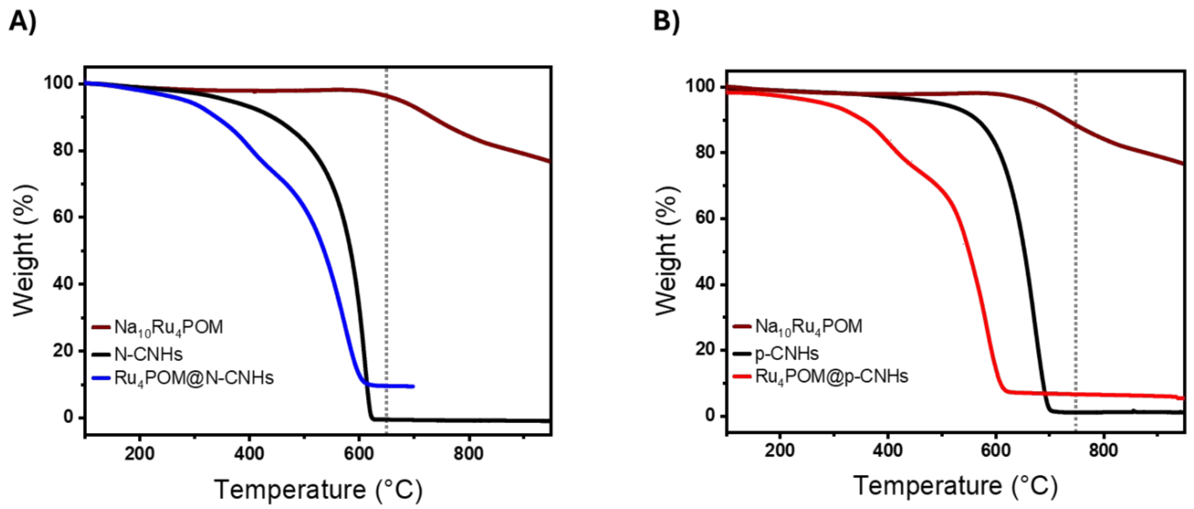

Figure S29. A. TGA analysis under air flow of $Na_{10}Ru_4POM$ (brown line), N-CNHs (black line), and Ru_4POM@N-CNHs (blue line). The remaining weight at 650 °C was used for estimating the loading of Ru_4POM on Ru_4POM@N-CNHs; **B.** TGA analysis under air flow of $Na_{10}Ru_4POM$ (brown line), p-CNHs (black line), and Ru_4POM@p-CNHs (red line). The remaining weight at 750 °C was used for estimating the loading of Ru_4POM on Ru_4POM@N-CNHs.

The loading of Ru_4POM on Ru_4POM@N-CNHs and Ru_4POM@p-CNHs was estimated with TGA. The N-CNHs and p-CNHs are completely degraded at 650 °C and 750°C respectively. So, in the hybrids Ru_4POM@N-CNHs and Ru_4POM@p-CNHs the remaining mass, at those temperatures, is expected to origin exclusively from the Ru_4POM^{10-} derivative. Considering the weight loss of $Na_{10}Ru_4POM$, the mass of Ru_4POM@N-CNHs at 650 °C and of Ru_4POM@p-CNHs at 750 °C were corrected accordingly, neglecting Na counterions, since, no Na was found in XPS spectra, as shown in **Figure S27F**. Utilization of the molar mass of Ru_4POM^{10-} (5459.26 g mol^{-1}) leads to a loading of 17.6 nmol mg^{-1} for Ru_4POM@N-CNHs and of 13.4 nmol mg^{-1} for Ru_4POM@p-CNHs. The earlier onset of weight loss in the hybrids compared to N-CNHs and p-CNHs is proposed to be due to the presence of Ru_4POM acting as a thermo-oxidation catalyst, according to literature.[26]

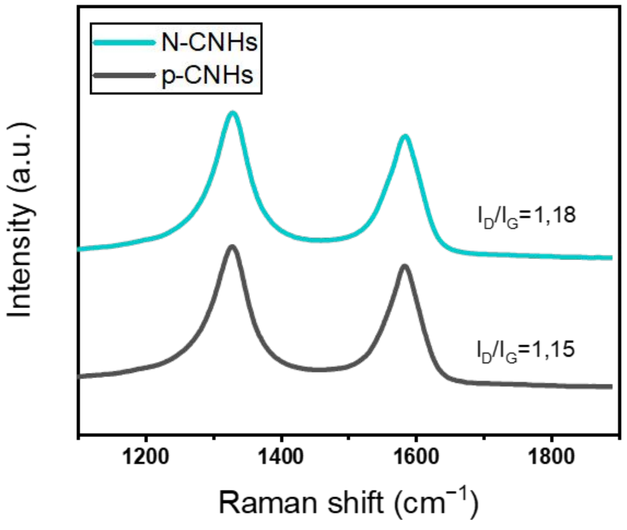

Figure S30. Stacked Raman spectra of p-CNHs (dark grey line) and N-CNHs (cyan line). The Raman spectra reveal the typical nanocarbon D and G bands of defective and crystalline sites, located respectively at 1333,6 cm-1 and 1582,5 cm-1. The intensity ratio of the D and G bands (ID/IG) increases from 1,15 to 1,18 after doping, and it is consistent with the introduction of defects due to the nitrogen doping process.27,28

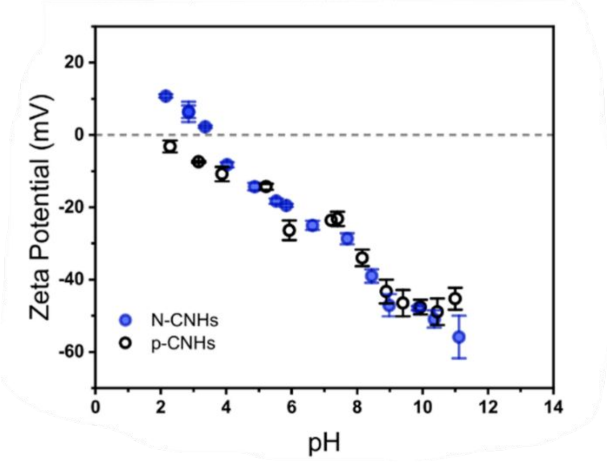

Figure S31. Zeta potential measurement of p-CNHs (black open circles) and N-CNHs (blue circles). The materials were dispersed in Milli-Q® water and titrated with HCl and KOH. A positive Zeta potential value, which means a slight stabilization of the colloids due to protonation, is emerging for N-CNHs at pH < 3.5. So, N-doping can favor the positive polarization of the CNHs surface and complementary electrostatic attraction of Ru_4POM.

Electrochemical surface area estimation by double layer capacitance

The C_{dl} was calculated as described in the Experimental Section. In this work the C_{dl} values were used to compare different electrodes in order to address variability in current density was observed across different materials batches, composites and electro-adsorbed anodes (**Figure S33, S37**). The issue of reproducibility may be attributed to the deposition method (drop casting), followed by solvent evaporation, and to electro-adsorption/desorption equilibria. This can lead to variations in the amount of electrochemically active material on the electrode surface, thereby impacting performance. the C_{dl} values were used for determining the electrode ESCA under optimized electro-adsorption conditions (see Experimental Section). So, the current outputs showed in **Figure S33** were normalized by the corresponding C_{dl} and the results are reported in **Figure S34,** confirming that the catalytic activity remains consistent across different materials samples. This analysis shows that Ru_4POM@N-CNHs and Ru_4POM@p-CNHs heterogeneous electrocatalysts display similar activity under CV and CPE conditions (**Figures S34, S35**).

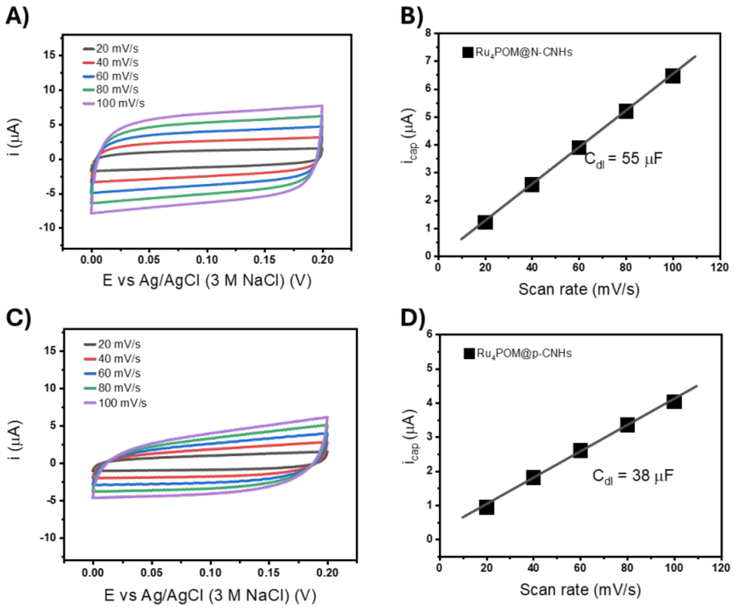

Figure S32: A, C. CVs at 20, 40 60 ,80 and 100 mV s^{-1} scan rate of an electrode of Ru_4POM@N-CNHs and Ru_4POM@p-CNHs respectively; **B, D.** i_{cap} vs. scan rate linear fit of Ru_4POM@N-CNHs and Ru_4POM@p-CNHs respectively.

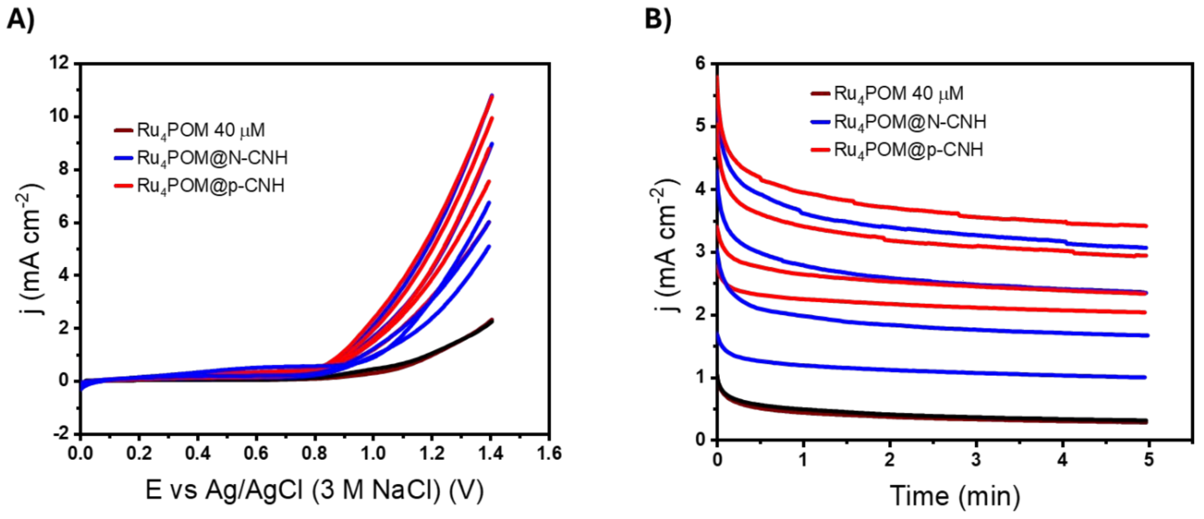

Figure S33: A. Linear sweep voltammetries (LSVs) of 4 batches of GCE modified with Ru_4POM@N-CNHs (blue line), Ru_4POM@p-CNHs (red line) and homogeneous Ru_4POM 40 µM (black line) for comparison; **B.** 5 minutes chronoamperometry (CA) at 1.2 V vs. Ag/AgCl of the same electrodes; the experiments were performed in EtOH, H_2O 4% v/v and $LiClO_4$ 0.2 M electrolytic solution; LSV scan rate 100 mV s^{-1}.

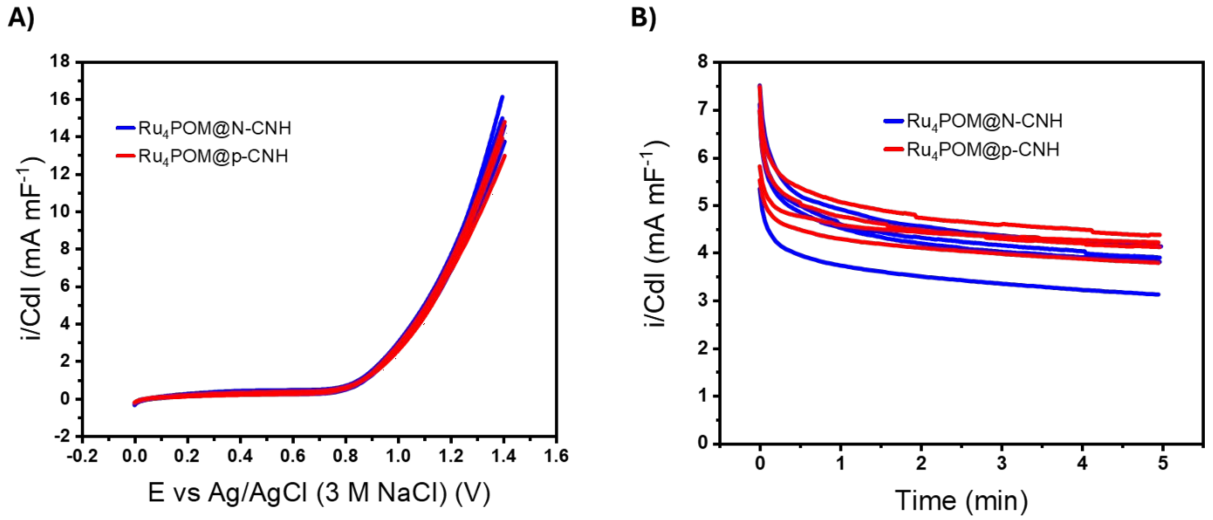

Figure S34: A. LSVs of 4 batches of GCE modified with Ru_4POM@N-CNHs (blue line), Ru_4POM@p-CNHs (red line) normalized for the corresponding C_{dl} **B.** 5 minutes CA at 1.2 V vs. Ag/AgCl of the same electrodes normalized for the corresponding C_{dl}; Ru_4POM@N-CNHs: 55 µF, 43 µF, 22 µF, 28 µF respectively; Ru_4POM@p-CNHs: 47 µF, 58 µF, 38 µF, 43 µF respectively; the experiments were performed in EtOH, H_2O 4% v/v and $LiClO_4$ 0.2 M electrolytic solution; LSV scan rate 100 mV s^{-1}.

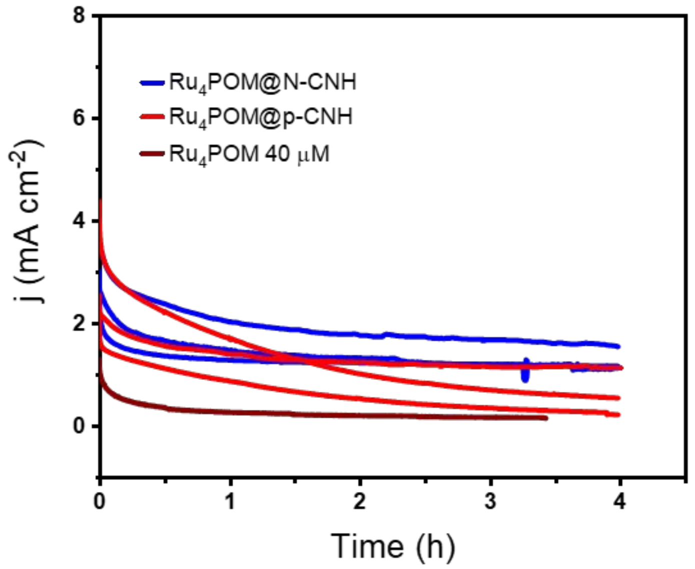

Figure S35: CPEs at 1.2 V vs Ag/AgCl over 4h of 3 samples of Ru_4POM@N-CNHs modified GCE (blue line), 3 samples of Ru_4POM@p-CNHs modified GCE (red line), homogeneous Ru_4POM 40 µM (brown line) with bare GCE as WE as reference, the experiments were performed in EtOH, H_2O 4% v/v and $LiClO_4$ 0.2 M electrolytic solution.

Electro-adsorption protocol for *in-situ* activation of the $Ru_4POM@CNHs$ interface.

The $Ru_4POM@CNHs$ interfaces is expected to undergo dynamic equilibria, due to Ru_4POM dissolution and deposition phenomena occurring between the electrode surface and the electrolyte. In particular the release of the Ru_4POM active clusters in the electrolyte solution leads to a progressive performance loss of the working electrode, as monitored by consecutive cyclic voltammetry (CV) experiments, addressing the electrode response under operation conditions (**Figure S36 A-D**). Controlled polarization of the nanostructured electrode can leverage the CNHs incorporation of Ru_4POM from the electrolyte, that mitigates the loss of active sites and leads to a continuous regeneration of the electrocatalytic surface. This concept refers to the surface activation via electro-adsorption and it has been recently highlighted for the dynamic polarization control of Ni electrodes for the oxygen evolution reaction (OER) (ref Nature Commun. 2025, https://www.nature.com/articles/s41467-025-60201-w). CNHs-based electrodes display some key properties for the electroadsorption protocol, namely: high specific surface area (up to 289 cm^2/mg, see equation 9-10), and electrical double-layer capacitance (up to 90 μF, **Figure S37**) (ref https://pubs.rsc.org/en/content/articlehtml/2023/ra/d2ra08252g).

CNHs modified GCE were prepared by drop-casting of a p-CNHs or N-CNHs ink (1 mg/mL in MeOH **Figure 5**) as described in the Equipment and Methods section, dipped within the EtOH 96% electrolytic solution containing $LiClO_4$ 0.2 M and Ru_4POM 40 μM. Electro-adsorption of Ru_4POM on the CNHs-GCE surface was driven upon application of positive voltage potentials chosen within a non faradaic region, with the aim to favor the electrostatic confinement of polyanionic Ru_4POM within the polarized CNH-based scaffold. Thus, Ru_4POM electro-adsorption was performed by continuous electrode polarization at +0.5 V vs. Ag/AgCl for 30 minutes. The resulting E-ads/$Ru_4POM@CNHs$ were used as working electrodes in a three electrode cell with a Ag/AgCl (NaCl 3M) as reference and a Pt wire as counter electrode. The electrodes were characterized by CV, CA and EIS experiments (**Figure S36, S37**).

CV experiments were performed by varying the scan rate to address the double-layer capacitance (C_{dl}) of electrodes A remarkable increase of C_{dl}, and thus of the electrochemically active surface area (ECSA see equation 9, 10) is observed upon Ru_4POM electro-adsorption whereby C_{dl} increases from 5 to 30 μF (**Figure S37 A, B**) for E-ads/Ru_4POM@p-CNHs, and from 20 to 90 μF for E-ads/Ru_4POM@N-CNHs (**Figure S37 C, D**). This indicates that the amount of charge in the electrochemical double layer is increased up to six folds after applying +0.5 V vs. Ag/AgCl for 30 minutes, due to Ru_4POM electro-adsorption under voltage-gated conditions, which leads to the significant increase in catalytic activity with respect to the POM-free scaffolds and to the homogeneous Ru_4POM reference (**Figure S36E**).

Chronoamperometry CA experiments performed under controlled potential electrolysis (CPE, at 1.2 V vs. Ag/AgCl applied voltage, **Figure S36F**) confirm that electrocatalysis is efficiently boosted by E-ads/$Ru_4POM@CNHs$, leading to enhanced current densities (up to 4 mA cm-2) and negligible loss across a short-time range (5 min) due to a favorable regeneration of the electrocatalytic surface by electroadsorption of Ru_4POM (40 μM) from the electrolyte.[30]

Inspection of the distribution of current density outputs depending on the CNHs batch (including both N- or p-CNHs) and on the electrode deposition series (**Figure S33-S35**) shows a ca. 26% average dispersion, indicating that similar responses are obtained for diverse $Ru_4POM@CNHs$ electrode series by using either N- or p-CNHs as scaffolds. Therefore, the long-term (18 h) CPE electrolysis experiment has been optimized using p-CNHs scaffolds under electro-adsorption conditions, as this offers a sustainable trade-off choice in terms of the best performance-to-cost ratio, no manufacturing process of the nanocarbon scaffold, self-activation of the electrocatalytic surface, similar mass-activity, life-cycle, stability and selectivity compared to the N-doped analogues (**Table S7 and S8**).

The average performance (ca. 26% yellow area) of E-ads/Ru_4POM@p-CNHs electrodes is highlighted in **Figure S38**, showing the long-term CPE experiment with plateau current densities in the range 2.2 -1.2 mA cm^{-2} and FE(EtOAc) > 70% (72 - 75 %) after 18 h electrolysis.

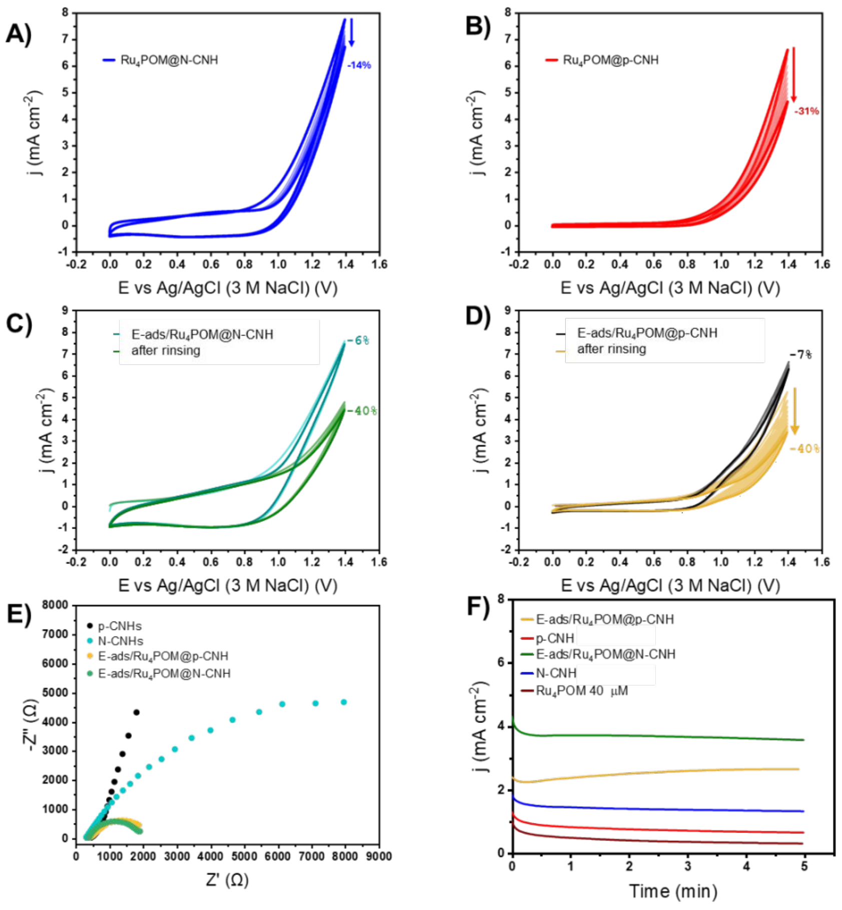

Figure S36: A. B. 10 consecutive CV of heterogeneous Ru_4POM@CNHs electrodes prepared by a drop-casting protocol and showing a loss of performance due to the release of the Ru_4POM clusters within the POM-free electrolyte solution. All experiments were performed in EtOH, H_2O 4% v/v and $LiClO_4$ 0.2 M electrolytic solution; CV scan rate 100 mV s^{-1}; the % current loss between the 1st and the 10th cycles was calculated at +1.2 V vs. Ag/AgCl yielding **A.** -14% for Ru_4POM@N-CNHs and **B.** – 31% for Ru_4POM@p-CNHs. **C. D.** 10 consecutives CVs of electro-adsorbed E-ads/Ru_4POM@CNHs electrodes prepared by a 30 min dipping of a CNH modified GCE within the same electrolyte solution, in the presence of Ru_4POM (40 µM) and under an applied voltage of +0.5 V vs. Ag/AgCl, showing the % current loss between the 1st and the 10th cycles, calculated at +1.2 V vs. Ag/AgCl yielding **C.** – 6% for E-ads/Ru_4POM@N-CNHs (cyan line) and **D.** -7% for E-ads/Ru_4POM@p-CNHs. The same electrode was removed from the electrodeposition solution, then rinsed, dried and probed again by 10 consecutives CVs in a fresh, POM-free, electrolytic solution, showing the % current loss between the 1st and the 10th cycles, calculated at +1.2 V vs. Ag/AgCl of > - 40% for **C.** E-ads/Ru_4POM@N-CNHs (green lines) and **D.** E-ads/Ru_4POM@p-CNHs. **E.** EIS characterization by Nyquist plot of p-CNHs modified GCE (black dots), N-CNHs modified GCE (cyan dots), E-ads/Ru_4POM@p-CNHs (yellow dots), E-ads/Ru4POM@N-CNHs (green dots); all measurements were performed in EtOH, H_2O 4% v/v and $LiClO_4$ 0.2 M electrolytic solution at an applied potential of +1.2 V vs. Ag/AgCl. **F.** CA experiments performed at 1.2 V vs. Ag/AgCl applied voltage, in a EtOH, H_2O 4% v/v and $LiClO_4$ 0.2 M, Ru_4POM (40 µM) electrolytic solution showing the variation of current density (j, mA cm^{-2}) over time (5 min) for E-ads/Ru_4POM@N-CNHs (blue line); E-ads/Ru_4POM@p-CNHs (yellow line) and control experiment performed with N-CNHs modified GCE (blue line), p-CNHs modified GCE (red line) and homogeneous Ru_4POM using a polished GCE (brown line).

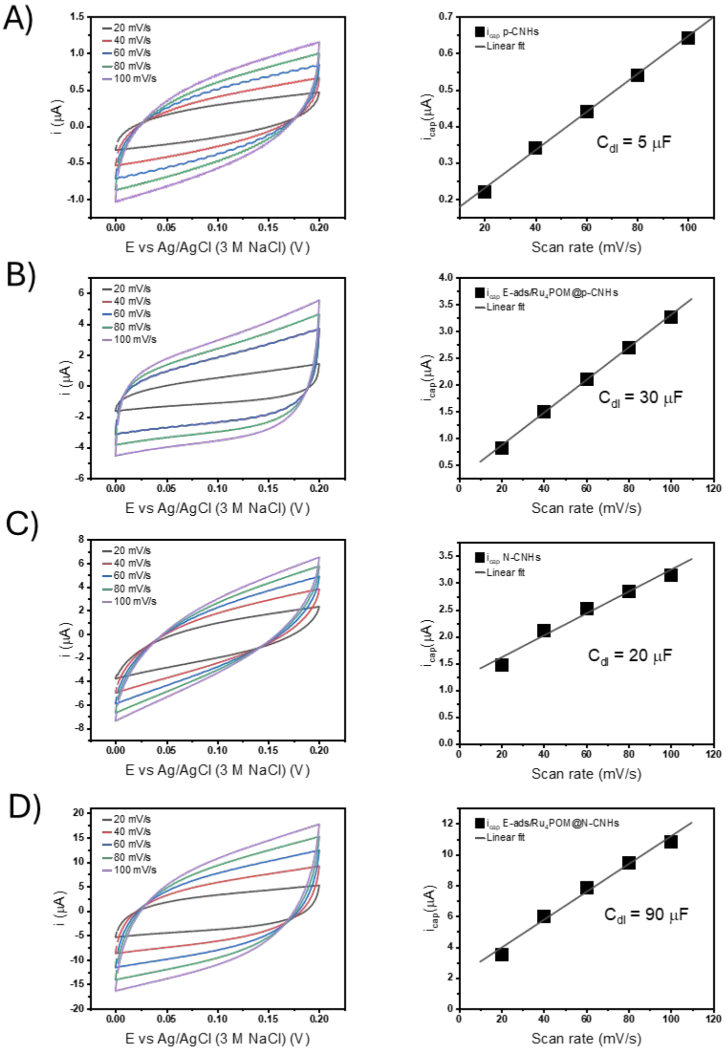

Figure S37: Determination of the double-layer capacitance (Cdl) by CV experiments recorded between 0 and 0.2 V vs. Ag/AgCl at scan rates of 20, 40, 60, 80 and 100 mV s^{-1} (left images) and linear fit of the capacitive current (i_{cap}) vs. scan rate with calculated C_{dl} (plots on the right panel) of: **A.** p-CNHs (R^2 = 0.95); **B.** E-ads/Ru_4POM@p-CNHs (R^2 = 0.99); **C.** N-CNHs (R^2 = 0.97); **D.** E-ads/Ru_4POM@N-CNHs (R^2 = 0.99).

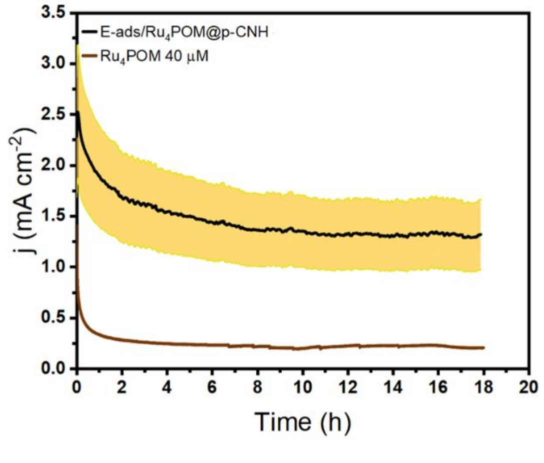

Figure S38: CPE performed at +1.2 V vs. Ag/AgCl over 18 h showing the variation of current densities over time that are registered for E-ads/Ru_4POM@p-CNHs (black line) in EtOH, H_2O 4% v/v and $LiClO_4$ 0.2 M, Ru_4POM (40 μM) electrolytic solution. The relative average dispersion of current density values (ca. 26% yellow area) is highlighted which stems from the current density distribution across diverse materials and electrodes batches (see Figure S33B). FE(EtOAc) is obtained in the range 72 - 75 % after 18 h electrolysis CPE by homogeneous Ru_4POM using a polished GCE (brown line) is reported as reference (brown line).

Table S7: Catalytic performance of E-ads/Ru_4POM@N-CNH and E-ads/Ru_4POM@p-CNH (Protocol C).

	TON	EtOAc yield (µmol cm^{-2})	TOF (s^{-1})	Mass activity (A g^{-1})	ECSA (cm^2 mg^{-1})
**E-ads/Ru_4POM@N-CNH**	**21165**	**53.2**	**1.47**	**2572**	**289**
**E-ads/Ru_4POM@p-CNH**	**19744**	**49.6**	**1.37**	**2271**	**263**

*The values of TON, TOF and mass activity reported in Table S7 were calculated according to the equations 4, 5, 7, 8 of the SI assuming the catalyst loading estimated by TGA (Figure S29); TON, TOF and EtOAc yield were calculated after 4 h of CPE at +1.2 V vs Ag/AgCl in EtOH, H_2O 4% v/v, $LiClO_4$ 0.2 M Ru_4POM 40 µM electrolytic solution, as described in Figure 6B. ECSA were calculated according to equations 9 and 10.

Table S8. Literature examples of selective partial electro-oxidation of ethanol and methanol.

Catalyst	Electrolyte/Solvent	Catalyst loading	Onset potential (V vs. SHE)	Peak potential (V vs. SHE)	CPE time (h)	FE % (product)	Ref.
Ru_4POM/PDDA	H_2SO_4 0.5 M/EtOH	n.r.	+1.25	+1.45	20	38.5 (acetaldehyde)<br>61.5 (AcOH)	12
Ru_4POM/PDDA	H_2SO_4 0.5 M/MeOH	n.r.	+1.06	+1.26	18.5	22.4 (formaldehyde)<br>77.6 (formic acid)	12
CpRu(CO)(µ-I)(µ-dppm)PtI_2 (homogeneous)	0.1 M TBAT/EtOH	10 mM	n.r.	+1.70	48	22 (diethoxyethane)	31
CpRu(CO)(µ-I)(µ-dppm)PtI_2 (homogeneous)	0.1 M TBAT/MeOH	10 mM	n.r.	+1.50	48	47 (dimethoxymethane)<br>16 (methyl formate)	31
CpRu(CO)(µ-I)(µ-dppm)PtI_2 (homogeneous)	0.1 M TBAT/MeOH (0.3% water)	10 mM	n.r.	+1.50	24	31 (dimethoxymethane)<br>16 (methyl formate)	31
Pt/C	Nafion membrane/Pure EtOH	0.972 mg (1.62 mg cm^{-2}) Pt	+0.50	+2.00	12	70 (diethoxyethane)	32
Pt/C	Pure MeOH	3 µg (15 µg cm^{-2}) Pt	+0.80	+1.90	24	80 (methyl formate)	14
β-FeOOH_F doped	15:5 EtOH:H_2O v:v	n.r.	+1.50	+1.59	12	72 (AcOH)	33
Pt NP	0.5 M $HClO_4$/0.1 M EtOH (pH 0.32)	n.r.	+0.68	+0.88<br>+1.23	n.r.	n.r. (acetaldehyde, AcOH)	34
Pt NP	0.5 M $NaClO_4$ 0.1 mM $HClO_4$/0.1 M EtOH (pH 3.92)	n.r.	+0.52	+0.67<br>+1.05	n.r.	n.r. (acetaldehyde at +0.67 V, acetaldehyde and AcOH at +1.05 V)	34
Pt NP	0.5 M $NaClO_4$/0.1 M EtOH (pH 6.16)	n.r.	+0.42	+0.56<br>+0.94	n.r.	n.r. (acetaldehyde at +0.56 V, acetaldehyde and AcOH at +0.94 V)	34
Ru/$Ni(OH)_2$ nanosheets	KOH 1 M/EtOH 1 M	n.r.	+0.58	+0.74	2	n.r. (acetaldehyde)	35

$MoO_3/Ni(OH)_2$	NaOH 1M/MeOH 1 M	10.8 nmol/cm^{-2} Ni	+0.52	+0.67	80	~100 % (formate)	36
$LaCo_{1-x}Fe_xO_3$ perovskite	KOH 0.1 M/EtOH 1 M	20 at. % Fe	+0.67	+0.87	0.33	n.r. (acetate, acetaldehyde)	37
Ru_4POM (homogeneous)	$LiClO_4$ 0.2M/EtOH (4 % v/v H_2O)	40 µM	+1.33	+1.70	3.5	57±3 (EtOAc)	This work
Ru_4POM (homogeneous)	$LiClO_4$ 0.2M/MeOH	40 µM	+1.45	+1.80	4.5	79±5 (methyl formate)	This work
E-ads/Ru_4POM@p-CNHs	$LiClO_4$ 0.2M/EtOH (4 % v/v H_2O)	N/A	+1.15	N/A	4	90±5 % (EtOAc)	This work
E-ads/Ru_4POM@N-CNHs	$LiClO_4$ 0.2M/EtOH (4 % v/v H_2O)	N/A	+1.15	N/A	4	89±5 (EtOAc)	This work

Table S9. Selected examples on ethanol electrooxidation for fuel cells applications.

Catalyst	Electrolyte/Solvent	Catalyst loading	Onset potential (V vs. SHE)	Peak potential (V vs. SHE)	CPE time (h)	FE % (product)	Ref.
[Ni(II)(salen)$(OH)_2$]	NaOH 1 M/MeOH 0.1 M	n.r.	+0.60	+0.80	n.r.	n.r. (formate, CO_2)	38
$Pd_{86}Sn_{14}/C$	KOH 0.5 M/ EtOH 0.5 M	3 µg (5 µg cm^{-2}) Pd+Sn	-0.26	-0.03	2	n.r.	39
PdCo NTAs/CFC	KOH 1 M/EtOH 1 M	23.4 µg cm^{-2} Pd	-0.33	+0.17	0.15	n.r.	40
Pd@g-C_3N_4/GC	KOH 1 M/EtOH 1 M	6.4 µg cm^{-2} Pd	-0.51	+0.07	n.r.	n.r.	41
Pd/N&F–C	KOH 1 M/EtOH 1 M	8 µg cm^{-2} Pd	-0.43	+0.01	1.7	~100 (CO_2)	42
Pt-Mo-Ni NWs	H_2SO_4 0.5 M/EtOH 2 M	10.8 µg cm^{-2} Pt	+0.44	+0.84	0.4	n.r. (CO_2)	43
FePd−Fe_2O_3/MWNTs	KOH 1 M/EtOH 1 M	3.71 µg cm^{-2} Pd	-0.36	-0.06	0.27	n.r.	44
$LaCo_{1-x}Fe_xO_3$ perovskite	KOH 0.1 M/EtOH 1 M	20 at. % Fe	+0.67	+0.87	0.33	n.r. (acetate, acetaldehyde)	37

Table S10. Electrochemical performance of all the studied catalytic systems

	**Onset potential (V vs. Ag/AgCl)**	**j at 1.2 V vs. Ag/AgCl (mA cm^{-2})**	**Av. j 5 minutes CA (j loss) mA cm^{-2}**	**Av. j 4 hours CPE (j loss) mA cm^{-2}**	**FE_{EtOAc}**
Ru_4POM 40 µM (4% H_2O)	+1.13	0.93	0.39 (72%)	0.23 (90%)	57±3 %
Ru_4POM 40 µM (0% H_2O)	+1.27	0.7	0.35 (30%)	0.17 (74%)	81±4 %
Ru_4POM 40 µM (8% H_2O)	+1.16	1.45	0.82 (42%)	0.24 (89%)	46±2 %
Ru_4POM µM (20% H_2O)	+1.15	3.2	1.8 (32%)	0.5 (90%)	24±2 %
E-ads/Ru_4POM @N-CNHs (4% H_2O)	+0.95	4.15	3.67 (17%)	1.66 (30%)	90±5 %
E-ads/Ru_4POM @p-CNHs (4% H_2O)	+0.95	3.10	2.50 (11%)	1.33 (18%)	89±5 %
E-ads/Ru_4POM @N-CNHs (8% H_2O)	+0.92	3.50	3.77 (1%)	2.80 (38%)	64 ±3
E-ads/Ru_4POM @p-CNHs (8% H_2O)	+0.98	2.39	3.53 (7%)	1.78 (59%)	59±4
E-ads/Ru_4POM @N-CNHs (20% H_2O)	+0.92	3.56	3.77 (13%)	2.47 (39%)	54±3
E-ads/Ru_4POM @p-CNHs (20% H_2O)	+0.98	2.60	2.80 (-18%)	1.70 (34%)	42±6

References

(1) Pagura, C.; Barison, S.; Mortalò, C.; Comisso, N.; Schiavon, M. Large Scale and Low Cost Production of Pristine and Oxidized Single Wall Carbon Nanohorns as Material for Hydrogen Storage. *Nanoscience and Nanotechnology Letters* **2012**, *4* (2), 160–164. https://doi.org/10.1166/nnl.2012.1308.

(2) Schiavon, M. Device and Method for Production of Carbon Nanotubes, Fullerene and Their Derivatives. US7125525B2, October 24, 2006.

(3) Yang, C. M.; Kim, Y. J.; Endo, M.; Kanoh, H.; Yudasaka, M.; Iijima, S.; Kaneko, K. Nanowindow-Regulated Specific Capacitance of Supercapacitor Electrodes of Single-Wall Carbon Nanohorns. *J Am Chem Soc* **2007**, *129* (1), 20–21. https://doi.org/10.1021/ja065501k.

(4) Omija, K.; Hakim, A.; Masuda, K.; Yamaguchi, A.; Kobayashi, M. Effect of Counter Ion Valence and PH on the Aggregation and Charging of Oxidized Carbon Nanohorn (CNHox) in Aqueous Solution. *Colloids Surf A Physicochem Eng Asp* **2021**, *619*, 126552. https://doi.org/10.1016/j.colsurfa.2021.126552.

(5) Fairley, N.; Fernandez, V.; Richard‐Plouet, M.; Guillot-Deudon, C.; Walton, J.; Smith, E.; Flahaut, D.; Greiner, M.; Biesinger, M.; Tougaard, S.; Morgan, D.; Baltrusaitis, J. Systematic and Collaborative Approach to Problem Solving Using X-Ray Photoelectron Spectroscopy. *Applied Surface Science Advances* **2021**, *5*, 100112. https://doi.org/10.1016/j.apsadv.2021.100112.

(6) Schneider, C. A.; Rasband, W. S.; Eliceiri, K. W. NIH Image to ImageJ: 25 Years of Image Analysis. *Nat Methods* **2012**, *9* (7), 671–675. https://doi.org/10.1038/nmeth.2089.

(7) Sartorel, A.; Carraro, M.; Scorrano, G.; Zorzi, R. De; Geremia, S.; McDaniel, N. D.; Bernhard, S.; Bonchio, M. Polyoxometalate Embedding of a Tetraruthenium(IV)-Oxo-Core by Template-Directed Metalation of $[\gamma\text{-SiW}_{10}O_{36}]^{8-}$: A Totally Inorganic Oxygen-Evolving Catalyst. *J Am Chem Soc* **2008**, *130* (15), 5006–5007. https://doi.org/10.1021/ja077837f.

(8) Zhuang, Z.; Giles, S. A.; Zheng, J.; Jenness, G. R.; Caratzoulas, S.; Vlachos, D. G.; Yan, Y. Nickel Supported on Nitrogen-Doped Carbon Nanotubes as Hydrogen Oxidation Reaction Catalyst in Alkaline Electrolyte. *Nat Commun* **2016**, *7* (1), 10141. https://doi.org/10.1038/ncomms10141.

(9) Toma, F. M.; Sartorel, A.; Iurlo, M.; Carraro, M.; Parisse, P.; Maccato, C.; Rapino, S.; Gonzalez, B. R.; Amenitsch, H.; Da Ros, T.; Casalis, L.; Goldoni, A.; Marcaccio, M.; Scorrano, G.; Scoles, G.; Paolucci, F.; Prato, M.; Bonchio, M. Efficient Water Oxidation at Carbon Nanotube–Polyoxometalate Electrocatalytic Interfaces. *Nat Chem* **2010**, *2* (10), 826–831. https://doi.org/10.1038/nchem.761.

(10) Quintana, M.; López, A. M.; Rapino, S.; Toma, F. M.; Iurlo, M.; Carraro, M.; Sartorel, A.; Maccato, C.; Ke, X.; Bittencourt, C.; Da Ros, T.; Van Tendeloo, G.; Marcaccio, M.; Paolucci, F.; Prato, M.; Bonchio, M. Knitting the Catalytic Pattern of Artificial Photosynthesis to a Hybrid Graphene Nanotexture. *ACS Nano* **2013**, *7* (1), 811–817. https://doi.org/10.1021/nn305313q.

(11) Chiang, Y.; Kresge, A. J. Kinetics of Hydrolysis of Acetaldehyde Ethyl Hemiacetal in Aqueous Solution. *J Org Chem* **1985**, *50* (25), 5038–5040. https://doi.org/10.1021/jo00225a007.

(12) Liu, Y.; Zhao, S.-F.; Guo, S.-X.; Bond, A. M.; Zhang, J.; Zhu, G.; Hill, C. L.; Geletii, Y. V. Electrooxidation of Ethanol and Methanol Using the Molecular Catalyst $[\{Ru_4O_4(OH)_2(H_2O)_4\}(\gamma\text{-SiW}_{10}O_{36})_2]^{10-}$. *J Am Chem Soc* **2016**, *138* (8), 2617–2628. https://doi.org/10.1021/jacs.5b11408.

(13) Lee, J. S.; Kim, J. C.; Kim, Y. G. Methyl Formate as a New Building Block in C1 Chemistry. *Appl Catal* **1990**, *57* (1), 1–30. https://doi.org/10.1016/S0166-9834(00)80720-4.

(14) Kishi, R.; Ogihara, H.; Yoshida-Hirahara, M.; Shibanuma, K.; Yamanaka, I.; Kurokawa, H. Green Synthesis of Methyl Formate via Electrolysis of Pure Methanol. *ACS Sustain Chem Eng* **2020**, *8* (31), 11532–11540. https://doi.org/10.1021/acssuschemeng.0c02281.

(15) Shi, Q.; Wei, X.; Raza, A.; Li, G. Recent Advances in Aerobic Photo‐Oxidation of Methanol to Valuable Chemicals. *ChemCatChem* **2021**, *13* (15), 3381–3395. https://doi.org/10.1002/cctc.202100104.

(16) Guthrie, J. P. Carbonyl Addition Reactions: Factors Affecting the Hydrate–Hemiacetal and Hemiacetal–Acetal Equilibrium Constants. *Can J Chem* **1975**, *53* (6), 898–906. https://doi.org/10.1139/v75-125.

(17) Matanovic, I.; Artyushkova, K.; Strand, M. B.; Dzara, M. J.; Pylypenko, S.; Atanassov, P. Core Level Shifts of Hydrogenated Pyridinic and Pyrrolic Nitrogen in the Nitrogen-Containing Graphene-Based Electrocatalysts: In-Plane vs Edge Defects. *The Journal of Physical Chemistry C* **2016**, *120* (51), 29225–29232. https://doi.org/10.1021/acs.jpcc.6b09778.

(18) Dürr, R. N.; Stéphan, E.; Leroy, J.; Oswald, F.; Verhaeghe, B.; Jousselme, B. Efficient, Stable, and Solvent-Free Synthesized Single-Atom Catalysts: Carbonized Transition Metal-Doped ZIF-8 for the Hydrogen Evolution Reaction**. *ChemElectroChem* **2023**, *10* (14). https://doi.org/10.1002/celc.202300205.

(19) Zhang, C.; Fu, L.; Liu, N.; Liu, M.; Wang, Y.; Liu, Z. Synthesis of Nitrogen-Doped Graphene Using Embedded Carbon and Nitrogen Sources. *Advanced Materials* **2011**, *23* (8), 1020–1024. https://doi.org/10.1002/adma.201004110.

(20) Lee, G. Y.; Kim, I.; Lim, J.; Yang, M. Y.; Choi, D. S.; Gu, Y.; Oh, Y.; Kang, S. H.; Nam, Y. S.; Kim, S. O. Spontaneous Linker-Free Binding of Polyoxometalates on Nitrogen-Doped Carbon Nanotubes for Efficient Water Oxidation. *J Mater Chem A Mater* **2017**, *5* (5), 1941–1947. https://doi.org/10.1039/C6TA09306J.

(21) Wu, P.; Qian, Y.; Du, P.; Zhang, H.; Cai, C. Facile Synthesis of Nitrogen-Doped Graphene for Measuring the Releasing Process of Hydrogen Peroxide from Living Cells. *J Mater Chem* **2012**, *22* (13), 6402. https://doi.org/10.1039/c2jm16929k.

(22) Biesinger, M. C. Accessing the Robustness of Adventitious Carbon for Charge Referencing (Correction) Purposes in XPS Analysis: Insights from a Multi-User Facility Data Review. *Appl Surf Sci* **2022**, *597*, 153681. https://doi.org/10.1016/j.apsusc.2022.153681.

(23) Major, G. H.; Fairley, N.; Sherwood, P. M. A.; Linford, M. R.; Terry, J.; Fernandez, V.; Artyushkova, K. Practical Guide for Curve Fitting in X-Ray Photoelectron Spectroscopy. *Journal of Vacuum Science & Technology A* **2020**, *38* (6). https://doi.org/10.1116/6.0000377.

(24) Morgan, D. J. Resolving Ruthenium: XPS Studies of Common Ruthenium Materials. *Surface and Interface Analysis* **2015**, *47* (11), 1072–1079. https://doi.org/10.1002/sia.5852.

(25) Rahimnejad, S.; Hui He, J.; Pan, F.; Lee, X.; Chen, W.; Wu, K.; Qin Xu, G. Enhancement of the Photocatalytic Efficiency of WO_3 Nanoparticles via Hydrogen Plasma Treatment. *Mater Res Express* **2014**, *1* (4), 045044. https://doi.org/10.1088/2053-1591/1/4/045044.

(26) Villani, K.; Kirschhock, C. E. A.; Liang, D.; Van Tendeloo, G.; Martens, J. A. Catalytic Carbon Oxidation Over Ruthenium-Based Catalysts. *Angewandte Chemie International Edition* **2006**, *45* (19), 3106–3109. https://doi.org/10.1002/anie.200503799.

(27) Zhang, Z.; Peng, B.; Chen, W.; Lai, Y.; Li, J. Nitrogen-Doped Carbon Nanotubes with Hydrazine Treatment as Cathode Materials for Lithium-Oxygen Batteries. *Journal of Solid State Electrochemistry* **2015**, *19* (1), 195–200. https://doi.org/10.1007/s10008-014-2591-z.

(28) Khare, B.; Wilhite, P.; Tran, B.; Teixeira, E.; Fresquez, K.; Mvondo, D. N.; Bauschlicher, C.; Meyyappan, M. Functionalization of Carbon Nanotubes via Nitrogen Glow Discharge. *J Phys Chem B* **2005**, *109* (49), 23466–23472. https://doi.org/10.1021/jp0537254.

(29) Morales, D. M.; Risch, M. Seven Steps to Reliable Cyclic Voltammetry Measurements for the Determination of Double Layer Capacitance. *Journal of Physics: Energy* **2021**, *3* (3), 034013. https://doi.org/10.1088/2515-7655/abee33.

(30) Jordan, J. W.; Cameron, J. M.; Lowe, G. A.; Rance, G. A.; Fung, K. L. Y.; Johnson, L. R.; Walsh, D. A.; Khlobystov, A. N.; Newton, G. N. Stabilization of Polyoxometalate Charge Carriers via Redox-Driven Nanoconfinement in Single-Walled Carbon Nanotubes. *Angewandte Chemie International Edition* **2022**, *61* (8). https://doi.org/10.1002/anie.202115619.

(31) Serra, D.; Correia, M. C.; McElwee-White, L. Iron and Ruthenium Heterobimetallic Carbonyl Complexes as Electrocatalysts for Alcohol Oxidation: Electrochemical and Mechanistic Studies. *Organometallics* **2011**, *30* (21), 5568–5577. https://doi.org/10.1021/om101070z.

(32) Kawaguchi, D.; Ogihara, H.; Kurokawa, H. Upgrading of Ethanol to 1,1-Diethoxyethane by Proton-Exchange Membrane Electrolysis. *ChemSusChem* **2021**, *14* (20), 4431–4438. https://doi.org/10.1002/cssc.202101188.

(33) Chen, G.-F.; Luo, Y.; Ding, L.-X.; Wang, H. Low-Voltage Electrolytic Hydrogen Production Derived from Efficient Water and Ethanol Oxidation on Fluorine-Modified FeOOH Anode. *ACS Catal* **2018**, *8* (1), 526–530. https://doi.org/10.1021/acscatal.7b03319.

(34) You, X.; Han, J.; Del Colle, V.; Xu, Y.; Chang, Y.; Sun, X.; Wang, G.; Ji, C.; Pan, C.; Zhang, J.; Gao, Q. Relationship between Oxide Identity and Electrocatalytic Activity of Platinum for Ethanol Electrooxidation in Perchlorate Acidic Solution. *Commun Chem* **2023**, *6* (1), 101. https://doi.org/10.1038/s42004-023-00908-3.

(35) Ao, W.; Cheng, C.; Ren, H.; Fan, Z.; Yin, P.; Qin, Q.; Chen, Z.-N.; Dai, L. Heterostructured Ru/Ni$(OH)_2$ Nanomaterials as Multifunctional Electrocatalysts for Selective Reforming of Ethanol. *ACS Appl Mater Interfaces* **2022**, *14* (39), 45042–45050. https://doi.org/10.1021/acsami.2c13864.

(36) Cheng, H.; Dong, B.; Liu, Q.; Wang, F. Direct Electrocatalytic Methanol Oxidation on MoO_3/$Ni(OH)_2$: Exploiting Synergetic Effect of Adjacent Mo and Ni. *J Am Chem Soc* **2023**, *145* (49), 26858–26862. https://doi.org/10.1021/jacs.3c09399.

(37) Alkan, B.; Cychy, S.; Varhade, S.; Muhler, M.; Schulz, C.; Schuhmann, W.; Wiggers, H.; Andronescu, C. Spray-Flame-Synthesized $LaCo_{1-x}Fe_xO_3$ Perovskite Nanoparticles as Electrocatalysts for Water and Ethanol Oxidation. *ChemElectroChem* **2019**, *6* (16), 4266–4274. https://doi.org/10.1002/celc.201900168.

(38) Bott-Neto, J. L.; Martins, T. S.; Machado, S. A. S.; Ticianelli, E. A. Electrocatalytic Oxidation of Methanol, Ethanol, and Glycerol on $Ni(OH)_2$ Nanoparticles Encapsulated with Poly[Ni(*Salen*)] Film. *ACS Appl Mater Interfaces* **2019**, *11* (34), 30810–30818. https://doi.org/10.1021/acsami.9b08441.

(39) Du, W.; Mackenzie, K. E.; Milano, D. F.; Deskins, N. A.; Su, D.; Teng, X. Palladium–Tin Alloyed Catalysts for the Ethanol Oxidation Reaction in an Alkaline Medium. *ACS Catal* **2012**, *2* (2), 287–297. https://doi.org/10.1021/cs2005955.

(40) Wang, A.; He, X.; Lu, X.; Xu, H.; Tong, Y.; Li, G. Palladium–Cobalt Nanotube Arrays Supported on Carbon Fiber Cloth as High-Performance Flexible Electrocatalysts for Ethanol Oxidation. *Angewandte Chemie International Edition* **2015**, *54* (12), 3669–3673. https://doi.org/10.1002/anie.201410792.

(41) Li, Z.; Lin, R.; Liu, Z.; Li, D.; Wang, H.; Li, Q. Novel Graphitic Carbon Nitride/Graphite Carbon/Palladium Nanocomposite as a High-Performance Electrocatalyst for the Ethanol Oxidation Reaction. *Electrochim Acta* **2016**, *191*, 606–615. https://doi.org/10.1016/j.electacta.2016.01.124.

(42) Chang, J.; Wang, G.; Wang, M.; Wang, Q.; Li, B.; Zhou, H.; Zhu, Y.; Zhang, W.; Omer, M.; Orlovskaya, N.; Ma, Q.; Gu, M.; Feng, Z.; Wang, G.; Yang, Y. Improving Pd–N–C Fuel Cell Electrocatalysts through Fluorination-Driven Rearrangements of Local Coordination Environment. *Nat Energy* **2021**, *6* (12), 1144–1153. https://doi.org/10.1038/s41560-021-00940-4.

(43) Mao, J.; Chen, W.; He, D.; Wan, J.; Pei, J.; Dong, J.; Wang, Y.; An, P.; Jin, Z.; Xing, W.; Tang, H.; Zhuang, Z.; Liang, X.; Huang, Y.; Zhou, G.; Wang, L.; Wang, D.; Li, Y. Design of Ultrathin Pt-Mo-Ni Nanowire Catalysts for Ethanol Electrooxidation. *Sci Adv* **2017**, *3* (8). https://doi.org/10.1126/sciadv.1603068.

(44) Wang, Y.; He, Q.; Guo, J.; Wang, J.; Luo, Z.; Shen, T. D.; Ding, K.; Khasanov, A.; Wei, S.; Guo, Z. Ultrafine FePd Nanoalloys Decorated Multiwalled Cabon Nanotubes toward Enhanced Ethanol Oxidation Reaction. *ACS Appl Mater Interfaces* **2015**, *7* (43), 23920–23931. https://doi.org/10.1021/acsami.5b06194.
